# Static and dynamic components of Debye–Waller coefficients in the novel cubic polymorph of low-temperature disordered Cu_2_ZnSnS_4_

**DOI:** 10.1107/S2052252522000239

**Published:** 2022-02-11

**Authors:** Eleonora Isotta, Binayak Mukherjee, Sebastian Bette, Robert Dinnebier, Paolo Scardi

**Affiliations:** a Department of Civil, Environmental and Mechanical Engineering, University of Trento, 77 via Mesiano, Trento 38123, Italy; b Max-Planck Institute for Solid State Research, Stuttgart Germany

**Keywords:** CZTS kesterite, hexagonal stacking faults, static and dynamic atomic meansquare displacement, Debye–Waller coefficients, ball-milling, structural disorder

## Abstract

Cu_2_ZnSnS_4_ (CZTS) is an attractive material for sustainable photovoltaics and thermoelectrics, and several properties originate from its marked polymorphism. High-energy mechanical alloying is found to lead to a disordered phase that possesses a sphalerite-like cubic structure. This is investigated in detail with the aid of laboratory and synchrotron radiation X-ray diffraction, Raman spectroscopy, electron microscopy and *ab initio* molecular dynamics. The disordered cubic polymorph is preserved below 663 K. With thermal treatments above 663 K, the tetragonal kesterite phase forms, used here as a reference for structural and microstructural features. Particular attention is paid to the stacking arrangement: a significant fraction of twin faults was found in the disordered cubic samples, which then progressively annealed with domain growth and with the transition to the ordered tetragonal phase. This study also focuses on Debye–Waller coefficients, which were found to be considerably larger for the disordered cubic than the tetragonal sample. Indeed, disorder leads to an ∼1 Å^2^ upward shift through the temperature range 100–700 K, a feature confirmed by *ab initio* calculations, which points to a particularly high contribution from disordered Sn cations. This supports the general understanding that structural disorder introduces a temperature-independent static contribution to the atomic mean-square displacement. Debye–Waller coefficients are found to be a good measure of this disorder, known to have a critical effect on transport properties.

## Abstract 2

Disorder greatly affects transport properties, as observed for Cu_2_ZnSnS_4_. A novel cubic polymorph of Cu_2_ZnSnS_4_ is characterized, and cation disorder is observed in a static contribution to Debye–Waller coefficients.

## 1. Introduction

Kesterite, with the reference formula Cu_2_ZnSnS_4_ (CZTS), is a sulfide mineral that has recently been the subject of intense investigation in different fields. It belongs to the family of *A*(I)_2_ *B*(II)*C*(IV)*X*(VI)_4_ quaternary compounds, with *A* = Cu, *B* = Zn, Fe, *C* = Sn and *X* = S, Se (Schorr, 2011 ▸). The term kesterite is typically used for the mineral, or by extension for the tetragonal *I* 4 crystal structure first associated with it (Hallt *et al.*, 1978 ▸). CZTS is a semiconducting chalcogenide, with a *p*-type conductivity arising from frequently occurring acceptor defects, such as Cu_Zn_ [predicted with the lowest formation energy (Walsh *et al.*, 2012 ▸)] and Cu_Sn_ antisites, and Cu vacancies (Siebentritt & Schorr, 2012 ▸). Owing to the direct band gap of ∼1.5 eV and high absorption coefficient on the order of 10^4^ cm^−1^ (Tanaka *et al.*, 2005 ▸), CZTS raised attention in the photovoltaic (PV) community as a possible absorber for thin-film solar cells. Indeed, it consists of earth-abundant, non-toxic and low-cost raw elements, thus representing an attractive alternative to other mature PV materials such as Cu(In, Ga)Se_2_. These encouraging premises, coupled with theoretical predictions of a conversion efficiency limit of 32.4% (Shockley & Queisser, 1961 ▸), led to extensive research on this material over the past 20 years (Wallace *et al.*, 2017 ▸). Despite the fact that CZTS is deemed the most promising PV material among the emerging critical-raw-material-free technologies (Giraldo *et al.*, 2019 ▸), to date the improvement has been rather feeble, with the best achieved efficiencies not exceeding 14% (Todorov *et al.*, 2010 ▸; Wang *et al.*, 2014 ▸; Son *et al.*, 2019 ▸; Yan *et al.*, 2018 ▸; Zhou *et al.*, 2021 ▸).

For the same reasons of sustainability and safety, CZTS has also been explored as a possible thermoelectric (TE) material (Liu *et al.*, 2009 ▸; Yang *et al.*, 2012 ▸; Kosuga *et al.*, 2015 ▸; Kumar *et al.*, 2018 ▸; Sharma & Neeleshwar, 2018 ▸; Zheng *et al.*, 2018 ▸; Nagaoka *et al.*, 2018 ▸; Isotta *et al.*, 2019*a* ▸,*b* ▸, 2020*a* ▸,*b* ▸, 2021*a* ▸; Sharma *et al.*, 2019 ▸, 2020 ▸; Jiang *et al.*, 2020 ▸; Long *et al.*, 2020 ▸; Baláž *et al.*, 2021 ▸). Given the large energy gap, CZTS shows an increasing performance with temperature, thus potentially appropriate for applications in the mid to high temperature range (600–800 K) (Kosuga *et al.*, 2015 ▸). Improved TE properties are obtained when doped with Cu (Yang *et al.*, 2012 ▸; Jiang *et al.*, 2020 ▸) or if S is replaced by Se (Liu *et al.*, 2009 ▸; Zheng *et al.*, 2018 ▸). Both bulk and thin-film (Kumar *et al.*, 2018 ▸; Isotta *et al.*, 2021 ▸ *b*) TE CZTS have been explored, showing an effective behaviour with particular kinds of defects and disorder (Isotta *et al.*, 2019*a* ▸, 2020*a* ▸,*b* ▸). Other investigations of CZTS include applications like tandem PVs (Todorov *et al.*, 2014 ▸; Giraldo *et al.*, 2019 ▸), photocatalysis (Ros *et al.*, 2018 ▸), photodetection (Wang *et al.*, 2011 ▸) and gas sensing (Gurav *et al.*, 2014 ▸; Shinde *et al.*, 2013 ▸). Recently, it has also been proposed as a possible candidates for hybrid thermoelectric photovoltaic solar harvesting (Narducci & Lorenzi, 2021 ▸).

**Figure 1:**
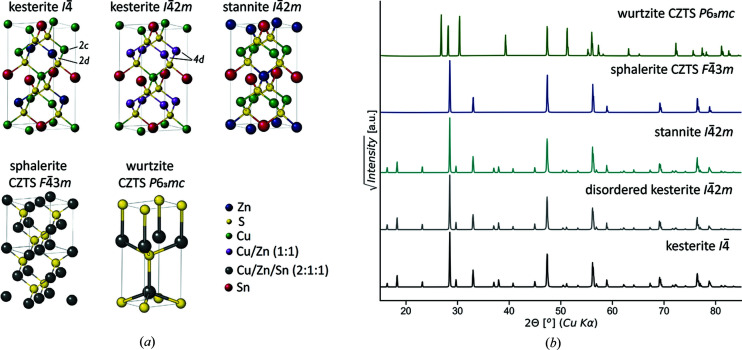
(*a*) Different crystal structures of Cu_2_ZnSnS_4_ (CZTS), from top left to bottom right: ordered tetragonal kesterite (space group *I* 4), disordered tetragonal (commonly called disordered kesterite, space group *I* 42*m*), stannite (space group *I* 42*m*), disordered cubic sphalerite CZTS (space group *F* 43*m*) and hexagonal wurtzite CZTS (space group *P*6_3_ *mc*). (*b*) XRD patterns for the different structures of CZTS, simulated for Cu *K*α_1_ radiation.

Owing to the chemical complexity, CZTS exhibits a pronounced polymorphism based on a tetrahedral coordination derived from the zinc-blende. The most commonly reported polymorph is the ordered tetragonal (space group *I* 4) kesterite-type structure. Another proposed crystallographic arrangement of CZTS is stannite (space group *I* 42*m*). Due to the low energy of formation of the Cu_Zn_ antisites, CZTS can also be found in the disordered tetragonal polymorph (space group *I* 42*m*, commonly referred to as disordered kesterite) described by Schorr *et al.* (2007 ▸). This was discovered to occur above 533 K, the order–disorder transition temperature, and frequently for lower temperatures, typically coexisting with the *I* 4 structure in metastable form, for kinetic reasons (Schorr & Gonzalez-Aviles, 2009 ▸; Scragg *et al.*, 2014*a* ▸; Schorr *et al.*, 2007 ▸). Disordered kesterite is characterized by full occupational disorder of Cu and Zn in the intermediate planes, that transforms 2*c* and 2*d* Wyckoff positions of *I* 4 into the unique 4*d* site of *I* 42*m* CZTS. The other Cu–Sn layer is instead identical for ordered and disordered kesterite. Stannite, despite possessing the same space group of disordered tetragonal kesterite, differs in the cation arrangement as it presents, perpendicularly to the *c* axis, alternating Cu-only and Sn–Zn layers. Fig. 1 ▸ shows the different crystal structures of CZTS and a simulation of their corresponding X-ray diffraction (XRD) patterns with Cu *K*α radiation. No major difference is observed between stannite, ordered and disordered kesterite. This is expected as they mainly differ in the arrangement of Cu^+^ and Zn^2+^ cations which, being isoelectronic, are indistinguishable using X-rays. *I* 4 kesterite was predicted by first-principle calculations as the most stable among these polymorphs, together with cation disorder in the Cu–Zn layer (Paier *et al.*, 2009 ▸). This was confirmed by neutron scattering measurements (Schorr, 2011 ▸; Schorr *et al.*, 2007 ▸), which can distinguish the two cations because of their different neutron scattering length. These, and other reported results (Chen *et al.*, 2009 ▸; Yu & Carter, 2015 ▸; Choubrac *et al.*, 2012 ▸) allowed it to be established that CZTS commonly arranges in the kesterite structure, ruling out stannite for stoichiometric conditions. CZTS seems to occur more frequently in a mixed ordered–disordered state, with the level of Cu–Zn disorder strongly dependent on the growth conditions and kinetics of thermal treatments. Further modifications of the tetragonal arrangement have been considered in the literature (Dimitrievska *et al.*, 2017 ▸; Paier *et al.*, 2009 ▸; Schorr, 2011 ▸).

Another reported polymorph of CZTS is the hexagonal *P*6_3_ *mc*, derived from the wurtzite ZnS structure. The phase is described as a hexagonally close-packed arrangement of sulfur atoms, with half the interstitial positions randomly occupied by Cu, Zn and Sn cations. This was first reported by Lu *et al.* (2011 ▸) and synthesized by hot-injection with the use of do­decane­thiol (DDT). Several other reports of wurtzite-type CZTS can be found, all reporting the use of DDT in the synthesis (Li *et al.*, 2014 ▸; Mainz *et al.*, 2014 ▸; Singh *et al.*, 2013 ▸, 2012 ▸; Zhou *et al.*, 2015 ▸; Lu *et al.*, 2011 ▸) and/or nanometre-dimensions (Aza­nza Ricardo *et al.*, 2015 ▸; Syafiq *et al.*, 2019 ▸). By cross-comparing results from different works, we noted a general higher abundance of wurtzite CZTS for grains with nanometre dimensions [<30 nm average dimensions (Li *et al.*, 2014 ▸; Mainz *et al.*, 2014 ▸; Singh *et al.*, 2013 ▸, 2012 ▸; Lu *et al.*, 2011 ▸; Zhou *et al.*, 2015 ▸; Aza­nza Ricardo *et al.*, 2015 ▸)], while, for increasing domain size, the hexagonal phase seems to reduce to stacking faults (Kattan *et al.*, 2015 ▸; Thompson *et al.*, 2016 ▸; Engberg *et al.*, 2020 ▸; Li *et al.*, 2014 ▸; Syafiq *et al.*, 2019 ▸) up to the point that neither (hexagonal arrangements nor faults) can be observed.

A cubic phase of CZTS has also been reported (space group *F* 43*m*) and is associated with the Sphalerite-type structure and represented as a cubic stacking of sulfurs in the [111] direction with a random occupation of the cation site by Cu, Zn and Sn. There seem to be two kinds of reports for this polymorph. The first is the high-temperature cubic (Schorr & Gonzalez-Aviles, 2009 ▸), which is the stable form of CZTS above 1156 K. High-temperature transitions to higher-symmetry structures are also found in other compounds of the adamantine family (Schorr & Geandier, 2006 ▸; Schorr *et al.*, 2006 ▸). The second is the metastable low-temperature cubic structure, obtained when the sample is synthesized via reactive mechanical alloying [*e.g.* by ball-milling (Kapusta *et al.*, 2019 ▸; Isotta *et al.*, 2019*b* ▸, 2020*b* ▸)], a production method gaining increasing interest as it is fast, simple and scalable. The highly disordered but low-temperature environment of high-energy mechanical alloying seems to favour a disordered arrangement of cations. Only a few reports on low-temperature cubic CZTS from ball milling can be found (Kapusta *et al.*, 2019 ▸; Isotta *et al.*, 2019*b* ▸, 2020*b* ▸), although in several works where kesterite was synthesized by mechanical alloying, the XRD patterns of the as-milled powders were clearly missing the tetragonal superstructure reflections (Ritscher *et al.*, 2016 ▸; Park *et al.*, 2014 ▸; Yao *et al.*, 2014 ▸; Pareek *et al.*, 2017 ▸; Hegedüs *et al.*, 2018 ▸; Long *et al.*, 2020 ▸; Baláž *et al.*, 2019 ▸). This was typically associated with disorder (Ritscher *et al.*, 2016 ▸) and, in rather loose terms, with low crystallinity (Shyju *et al.*, 2015 ▸; Zhou *et al.*, 2016 ▸; Zhang *et al.*, 2019 ▸; Alirezazadeh & Sheibani, 2020 ▸). Occasionally low-temperature sphalerite CZTS has been reported from hot-injection preparations as well (Brandl *et al.*, 2015 ▸), sometimes mixed with the wurtzite (Syafiq *et al.*, 2019 ▸) or the kesterite (Ahmad *et al.*, 2015 ▸; Engberg *et al.*, 2020 ▸) phases. The polymorph is metastable, as it is reported to transform into tetragonal kesterite above ∼663 K (Isotta *et al.*, 2020*b* ▸). Similar low-temperature sphalerite phases were reported for other multinary chalcogenides (Lohani *et al.*, 2020 ▸; Baláž *et al.*, 2021 ▸).

Disorder in kesterite seems to be of particular importance: from a TE perspective, Cu–Zn disorder is found to induce electronic band-degeneracy, remarkably improving the thermopower (Isotta *et al.*, 2019*a* ▸, 2020*a* ▸, 2021*a* ▸); full cation disorder is instead discovered to optimize all three TE parameters at the same time (Isotta *et al.*, 2020*b* ▸), namely suppressing the thermal conductivity and enhancing the electrical conductivity and Seebeck coefficient. On the other hand, disorder seems to negatively affect PV performance. Numerous investigations were devoted to the role of Cu–Zn disorder (Scragg *et al.*, 2014*b* ▸, 2016 ▸; Rudisch *et al.*, 2016 ▸; Valentini *et al.*, 2016 ▸; Mendis *et al.*, 2017 ▸; Schorr *et al.*, 2007 ▸; Schorr & Gonzalez-Aviles, 2009 ▸), whereas more recent studies point to extensive disorder and Sn_Zn_ antisites as the true source of PV efficiency loss (Chen *et al.*, 2021 ▸). In addition, recent theoretical studies predict that high cation disorder induces a transition to a topologically non-trivial phase, attributing disordered CZTS to the class of topological Anderson insulators (TAIs) (Mukherjee *et al.*, 2021*b* ▸).

It is therefore essential to further study and understand this kind of disorder. The aim of this work is to perform a structural characterization of the low-temperature cubic CZTS phase made by mechanical alloying and compare it with tetragonal kesterite. Particular attention is paid to the presence of faults in the stacking arrangement and morphological features. The temperature stability and evolution of the phases are investigated. A careful experimental and theoretical study of the temperature trend of the Debye–Waller (DW) coefficients allows us to identify static and dynamic components for the disordered cubic phase. This can contribute to a general understanding of the role of cationic disorder in the thermal behaviour of the material. Advancement in understanding disordered CZTS can shed light on important disorder-induced properties such as TAI behaviour, as well as the reported critical consequences on TE and PV performance.

## 2. Methods

### 2.1. Sample preparation

CZTS powders were synthesized via reactive mechanical alloying in a planetary mill (Fritsch P4 Pulverisette 4). Elemental precursors (Cu powder, <75 µm, 99%; Zn powder, purum, 99%; Sn powder, puriss, 99%, S flakes, purum, 99.5%; all from Sigma–Aldrich) were weighed in stoichiometric quantities with a ball-to-powder weight ratio of 100:1. The milling was performed with an 80 ml brass jar and 25 brass balls (12 mm in diameter) as milling medium. The whole procedure of vial filling, milling and powder collection was performed in air. A volume of 480 µl ethanol (99.8%, Sigma–Aldrich) was added to the precursor mixture as lubricant. High-energy milling conditions were used, with jar rotation ω = −540 rpm, main disk revolution Ω = 300 rpm, for a fixed ratio ω/Ω = −1.8 (Broseghini *et al.*, 2016 ▸; Isotta *et al.*, 2019*b* ▸), and a milling time of 60 min. This milling time was selected to guarantee minimal contamination from the vial and ball material (Isotta *et al.*, 2019*b* ▸). Thermal treatments of powders were performed to achieve the cubic or tetragonal structures. For cubic samples: 60 min at 433 K (heating rate *r* = 20 K min^−1^) followed by 20 min at 573 K (*r* = 20 K min^−1^ up to 533 K and 10 K min^−1^ from 533 to 573 K); for tetragonal samples: 60 min at 573 K (heating rate *r* = 20 K min^−1^) followed by 20 min at 833 K (*r* = 20 K min^−1^ up to 793 K and 10 K min^−1^ from 793 to 833 K). After treatment, the samples were left to naturally cool down to ambient temperature. Thermal treatments were performed in a tubular oven under Ar flux (estimated O_2_ level < 10 p.p.m.).

### 2.2. X-ray diffraction

High-resolution synchrotron radiation X-ray diffraction (SRXRD) was performed at the Paul Scherrer Institute (Villigen, Switzerland), MS Beamline X04SA. Data were collected with the Mythen II detector, at a wavelength of 0.5639 Å (∼22 keV). Measurements at room temperature and high temperature were performed. For the latter, isothermal measurements every 75 K were performed in the ranges 323–773 or 323–873 K. A temperature ramp of 10 K min^−1^ was provided by a hot air blower. Si640d standard patterns were collected to model the instrumental profile. Specimens were sealed in 0.3 mm-diameter quartz glass (for high temperature) and borosilicate glass (for room temperature) capillaries spun during the measurement. For measurements at high temperature intended to accurately estimate the DW coefficients, samples were diluted with 50%vol glass powder from ground capillaries. This was applied to decrease the X-ray absorption to the point where absorption correction is unnecessary (reaching a linear absorption coefficient μ*R* ≃ 0.3, such that systematic deviations in intensity between low and high angles are <1%). Low-temperature patterns were recorded on a StadiP powder diffractometer (Stoe) using Debye–Scherrer geometry and Ag *K*α_1_ radiation from a primary Ge(111)-Johann-type monochromator equipped with a triple array of Mythen 1 K (Dectris) detectors. The capillaries were heated to 400 K and subsequently cooled to 100 K using a hot and cold air blower (Cobra 700, Oxford Cryosystems) applying heating and cooling rates of 5 K min^−1^. XRPD patterns were taken in 50 K intervals applying a scan range from 0 to 110° 2θ, a scan time of 8 h and a delay time of 30 min prior to each measurement to ensure thermal equilibration.

Rietveld refinements (Rietveld, 1969 ▸) of XRD data were performed with the software *TOPAS* (version 7; Coelho, 2018 ▸). Crystallite size and strain analysis was carried out with the support of macros based on whole powder pattern modelling (WPPM) (Scardi & Leoni, 2002 ▸; Scardi *et al.*, 2018 ▸), directly including microstructural parameters for the refinement of the data. For size broadening, a lognormal distribution of spherical domains was considered, from which the arithmetic mean size 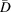 , standard deviation σ and volume-weighted mean column height *D*_v_ were calculated. The choice of a spherical shape for the crystal domain modelling was supported by transmission electron microscopy observations. For strain, the PAH model (from Popa, Adler and Houska, who provided phenomenological observations), deemed a flexible approach to model a general microstrain, was applied (Scardi *et al.*, 2018 ▸). For certain phases, in order to limit the number of free parameters, estimations of mean size and strain were performed with built-in *TOPAS* macros, based on the double-Voigt method (Dinnebier *et al.*, 2018 ▸). This approach was applied when the phases were present in small amounts and introducing WPPM would increase the number of refinable parameters making the fit unstable. Built-in *TOPAS* macros provide the average microstrain (*e*_0_) and the volume-weighted mean size based on the integral breadth (*L*_Vol_) from a combination of Gaussian and Lorentzian strain broadening. *L*_Vol_ is calculated assuming spherical domains and is conceptually comparable with *D*_v_. To be precise, they share the same definition, but the first is extracted from the modelling of peaks with Voigt functions, the latter introduces a lognormal distribution of spherical domains directly in the model.

Fault scenarios in cubic CZTS were evaluated using *TOPAS* software (Coelho, 2018 ▸) for the modelling of stacking fault disorder and for the determination of fault probabilities.

### 2.3. Electron microscopy

Scanning electron microscopy (SEM) imaging and corresponding energy-dispersive X-ray spectroscopy (EDXS) were performed with a Coxem EM-30AX instrument. Transmission electron microscopy (TEM) imaging, selected area electron diffraction (SAED) and high-magnification EDXS were performed with a high-resolution scanning/transmission electron microscopy instrument (ThermoFischer TALOS 200s).

### 2.4. Elemental composition

The elemental composition of the samples was analyzed by inductively coupled plasma (ICP) mass spectrometry for the determination of the metal contents and by combustion analysis (CHNS) for the sulfur content. For the ICP analysis the samples were dissolved in aqua regia in closed Ni-capsules to avoid extrusion of volatile Sn species. The capsules were heated in a microwave (CEM) at 180°C for 35 min. The ICP analysis was carried out using an ICP-OES spectrometer (Vista Pro) equipped with an axial plasma source, an Echelle-polychromator and a CCD detector (Agilent Technologies). For the analysis, the following emission lines were used: Cu 327.395 nm, Sn 283.998 nm and Zn: 206.200 nm. The analysis of the sulfur content was carried out using a Vario Micro Cube analyser (Elementar).

### 2.5. Raman spectroscopy

Raman spectra were recorded using a Jobin Yvon Typ V 010 LabRAM single-grating spectrometer, equipped with a double super razor edge filter and a Peltier-cooled charge-coupled device camera. The resolution of the spectrometer (grating, 1800 lines mm^−1^) was 1 cm^−1^. The spectra were taken in a quasi-backscattering geometry using the different linearly polarized lasers with wavelengths of 632 and 532 nm. The maximum power used was 4 mW to protect against local heating. The spot size was 5–10 µm, focused by a 100× microscope objective on the surface of the sample.

### 2.6. *Ab initio* molecular dynamics

The *ab initio* molecular dynamics simulations were performed using the *Vienna *ab initio* simulation package* (*VASP*) (Kresse & Furthmüller, 1996*a* ▸,*b* ▸). A 64-atom supercell was used to represent both the ordered tetragonal and disordered cubic polymorphs of CZTS. The electron-exchange correlation functional was approximated using the Perdew–Burke–Ernzerhof (PBE) (Perdew *et al.*, 1996 ▸) form of the generalized gradient approximation, with the scalar-relativistic PBEsol pseudopotentials (Perdew *et al.*, 2008 ▸) shown to be highly effective in calculating the elastic and mechanical properties of solids (Ahuja *et al.*, 2015 ▸; Terentjev *et al.*, 2018 ▸; Maschio *et al.*, 2011 ▸). All calculations were performed with an energy cutoff of 400 eV and a Gaussian charge smearing of 0.1 eV. The irreducible Brillouin zone was sampled with a 2 × 2 × 2 Monkhorst–Pack gamma-centred k-mesh, with electronic degrees of freedom relaxed until the changes in the total free energy and energy eigenvalues were both smaller than 10^−6^ eV. The molecular dynamics simulations were performed within a canonical (NVT) ensemble connected to a heat bath with a Nose–Hoover thermostat, set to 10, 100, 300, 500 and 700 K for multiple trajectories. In each case, the system was allowed to evolve with a timestep of 2 fs, for 10 000 steps, corresponding to a total simulation time of 20 ps. The trajectories were subsequently visualized using *OVITO* (Stukowski, 2010 ▸), and the root-mean-square displacement was calculated from the trajectories using *VMD* (Humphrey *et al.*, 1996 ▸). In order to measure the anisotropy, we calculated the interatomic force constants from density functional theory (DFT) via the finite difference method, from which we calculated the log-Euclidian anisotropy parameter (Kube, 2016 ▸).

## 3. Results and discussion

### 3.1. A zinc-blende derived disordered phase

**Figure 2:**
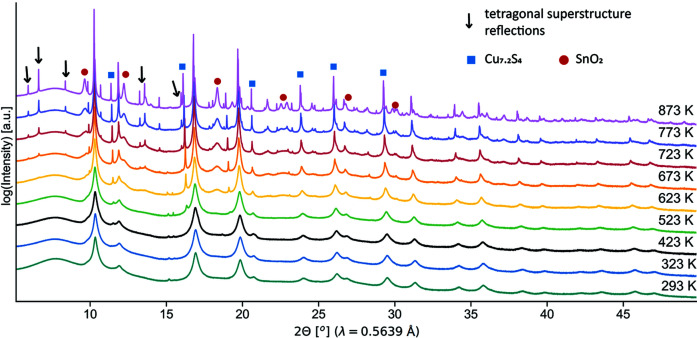
SRXRD over temperature for an as-milled CZTS sample, with indication of tetragonal superstructure reflections and secondary phases.

High-resolution SRXRD patterns are collected over the range 293–873 K on a sample of as-milled powder and can be seen in Fig. 2 ▸. As already highlighted in previous work from some of the authors (Isotta *et al.*, 2020*b* ▸), mechanical alloying appears to promote a disordered arrangement of cations leading to a cubic sphalerite-like *F* 43*m* crystal structure. Peaks are generally broad, pointing to small and defected domains, in agreement with TEM observations (see TEM imaging in Note S1 of the supporting information). This cubic phase appears preserved until 673 K, where additional peaks (see black arrows in Fig. 2 ▸) develop in the XRD patterns, hallmarking the transition to the lower symmetry phase of tetragonal kesterite. Thermal analyses by Isotta *et al.* (2020*b* ▸) also confirm this observation, locating the cubic-to-tetragonal CZTS transition at ∼663 K. Additional peaks in the higher-temperature SRXRD patterns are attributed to SnO_2_ and Cu_7.2_S_4_ secondary phases.

**Figure 3:**
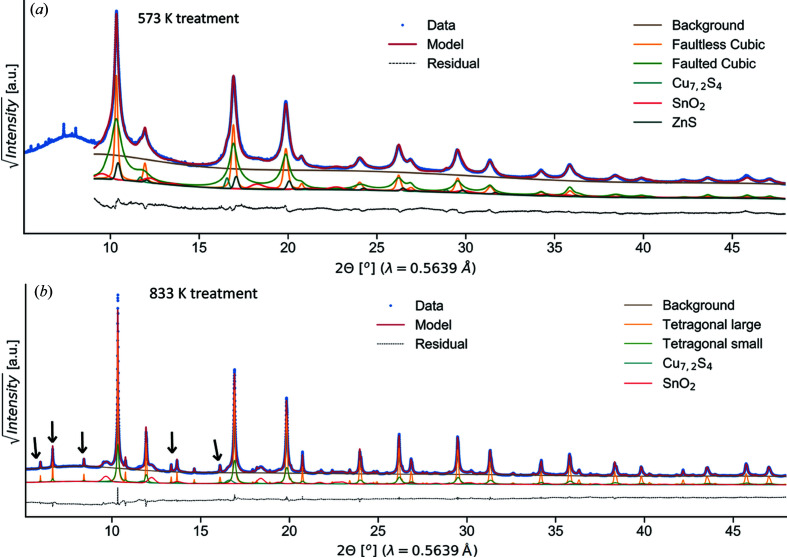
Synchrotron radiation X-ray diffraction data with Rietveld refinement for CZTS samples from mechanical alloying, thermally treated at 573 K (*a*) or 833 K (*b*) to stabilize the cubic or the tetragonal phase, respectively. Data are plotted as dotted blue, modelling as solid red and residual as dashed black. Models of individual phases are shown as coloured solid lines. Some of the most intense tetragonal superstructure reflections are marked by black arrows.

**Figure 4:**
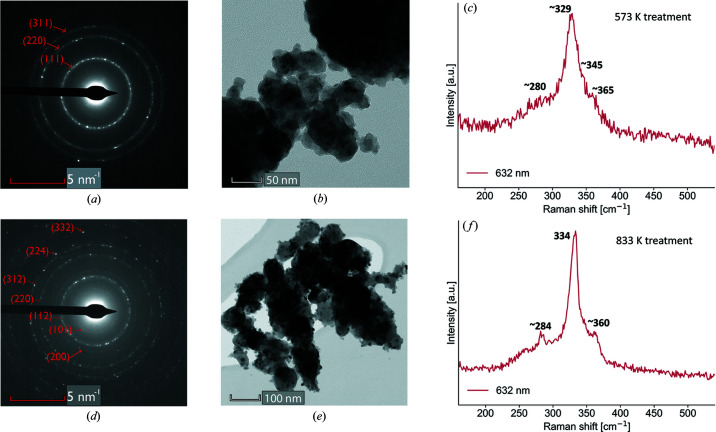
(*a*) and (*d*) Selected area electron diffraction patterns with indexing and (*b*) and (*e*) transmission electron microscopy images for CZTS samples from mechanical alloying, treated at (*a*) and (*b*) 573 K and (*d*) and (*e*) 833 K. Raman spectra for samples treated at (*c*) 573 K and (*f*) 833 K, measured with a 632 nm laser.

**Figure 5:**
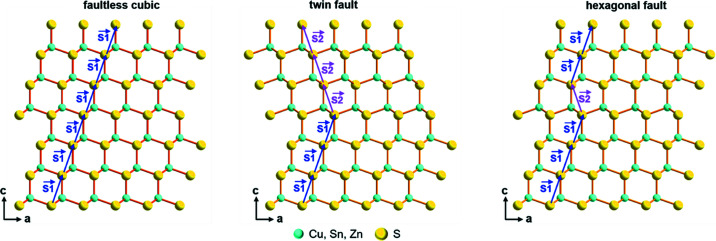
Structural motifs considered to occur in the microstructure of cubic CZTS. From left to right: faultless cubic stacking, cubic stacking with a twin fault, cubic stacking with a hexagonal deformation fault. 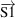 and 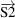 represents the stacking vectors.

To better understand this phase transition and the differences between the cubic and tetragonal phases of CZTS, a detailed structural investigation of two thermally treated samples was performed. Treatment temperatures were chosen below (at 573 K) and above (at 833 K) the transition temperature. SRXRD data and modelling with Rietveld refinement are presented in Fig. 3 ▸; fit parameters are reported in Table 1 and Fig. 5 and will be discussed in the next section. A thermal treatment at 573 K [Fig. 3 ▸(*a*)] seems to preserve the disordered cubic CZTS reflections observed for the as-milled powders, whereas at 833 K we observe the development of the tetragonal kesterite phase. To confirm the structural arrangement, SAED was performed [Figs. 4 ▸(*a*) and 4 ▸(*d*)]. Both samples present the same major reflections. Nevertheless, the SAED pattern of the sample treated at 833 K clearly displays additional lower-intensity rings. These are compatible with the tetragonal superstructure reflections, as indicated by the indexing. This suggests that also ‘locally’ the sample treated at 573 K lacks tetragonal ordering. We therefore propose the structure is derived from the zinc-blende, or sphalerite, with a random occupation of Cu, Zn and Sn in the cation site. Based on SEAD results, no evidence of medium-range order is noted.

High-magnification EDXS (see Note S1) and Raman spectroscopy [Figs. 4 ▸(*c*), 4 ▸(*f*) and Note S2] allowed us to ultimately rule out the possible formation of Cu_2_SnS_3_ (CTS) and ZnS. These, by virtue of sharing the same main reflections of CZTS, are hard to identify with XRD though totally compatible with the overall sample stoichiometry. All the CZTS elements were found with EDX on single domains. Furthermore, Raman spectra show the main modes of CZTS (Himmrich & Haeuseler, 1991 ▸; Altosaar *et al.*, 2008 ▸) whereas those of CTS (Fernandes *et al.*, 2010 ▸; Lohani *et al.*, 2020 ▸) and ZnS (Nilsen, 1969 ▸) can be excluded. Elemental composition was investigated with ICP mass spectrometry and CHNS analysis. Samples were found to be slightly Sn-poor and Zn-rich, while the composition of the other elements was close to stoichiometric. Further details can be seen in Note S1.

### 3.2. Occurrence of stacking faults: different scenarios

For the sample treated at 573 K and presenting the XRD reflections of disordered cubic CZTS, a multiphase modelling approach is utilized. Indeed, a single phase of sphalerite CZTS does not perfectly model the data, which shows shoulders at the base of the main peaks and diffuse scattering. These features could be explained by the presence of layer faulting. This assertion is backed up by the literature on CZTS (Kattan *et al.*, 2016 ▸, 2015 ▸; Brandl *et al.*, 2015 ▸; Engberg *et al.*, 2020 ▸; Ahmad *et al.*, 2015 ▸) and similar systems (Fonoll-Rubio *et al.*, 2021 ▸), highlighting the presence of twinning and/or alternating hexagonal and cubic stacking (depicted in Fig. 5 ▸). Therefore, we have introduced faulting models in the refinements. Stacks of 500 layers were randomly generated and their diffraction patterns calculated. For every refinement, 100 XRD patterns were averaged (Coelho *et al.*, 2016 ▸). A probability of transitioning to a faulted layer was introduced when generating the stacks. Multidimensional grid-search routines (Bette *et al.*, 2019 ▸, 2020 ▸) were employed to find global minima in the parameter space of the faulting probabilities. Further modelling details and results of the minimization can be found in Note S3. The selected microstructural model consists of two phases of cubic CZTS: one with and one without faults. Attempts to refine a unique faulted CZTS phase have been performed, but yielded some misfits and a significantly higher *R*_wp_ value. Faultless cubic CZTS is modelled as *F* 43*m* ZnS with a fractional occupation of the Zn site with Cu, Zn and Sn in the ratio 2:1:1. In the faulted CZTS phase, a probability of twin faults is introduced in the cubic stacking (where a single fault would appear as a twin, and perfect hexagonal stacking would yield a probability of 100%). *TOPAS* .str files for the modelling are visible in Note S4. The cell parameters of the two phases are imposed as equivalent (in the corresponding cubic system). Minimization shows a fault probability of 22% (corresponding to 1 fault per ∼1.5 nm). These observations are in reasonable agreement with the literature. Ahmad *et al.* (2015 ▸) found a coexistence of cubic and hexagonally faulted cubic CZTS in samples from hot-injection. Extensive twinning and stacking faults were observed by Thompson *et al.* (2016 ▸), where TEM imaging was used to assess a fault density of 1 per 10 nm. These were held responsible for a suppression in thermal conductivity. Domains were larger in their case thus supporting a progressive annealing of faults with grain growth. *Ab initio* calculations (see Note S5) also support this statement. Indeed, the sphalerite-like and the wurtzite-like CZTS structures are calculated, and found to possess similar values of ground-state binding energy, with the former slightly favoured energetically. This can support coexistence of the phases under certain conditions, with a tendency to evolve towards the cubic structure.

### 3.3. Microstructural features

**Figure 6:**
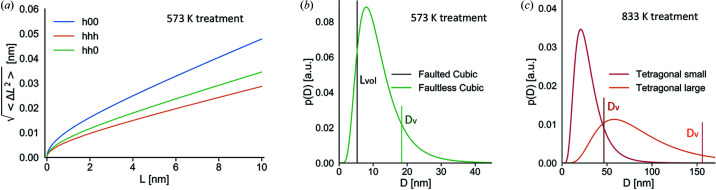
(*a*) Warren plot (Warren & Averbach, 1950 ▸) for (*h*00), (*hhh*) and (*hh*0) directions, yielding a graphical representation of microstrain, for the sample thermally treated at 573 K. It represents the standard deviation of the distribution of atomic displacement between couples of unit cells at increasing distance *L*, from zero to the maximum extension of the crystalline domains along the considered crystallographic direction. (*b*) and (*c*) Domain size distributions for both samples. For the phases modelled with WPPM, a lognormal distribution of spherical domains with size *D* is assumed (coloured solid lines), with volume-weighted mean column height *D*_v_ shown as a vertical line. The vertical black line in (*b*) indicates the volume-averaged mean size from integral breadth, *L*_Vol_, always considering spherical domains. See the main text for modelling details.

The results of the line profile analysis are reported in Table 1 ▸ and in the Warren plot (Warren & Averbach, 1950 ▸) of Fig. 6 ▸, which also shows the distribution of domain sizes. Crystallite size is estimated on the order of 5 to 20 nm, in reasonable accordance with TEM images reported in Fig. 4 ▸(*b*) and Note S1. The Warren plot for the faultless fraction predicts the [*hhh*] as the stiffer direction, in accordance with DFT results (see compliance tensors in Note S6). For the faulted fraction, the refinement yields *e*_0_ = 0. This result probably underestimates *e*_0_: strain broadening effects could instead be concealed in the faulting or have a strong correlation with other parameters.

Secondary phases are introduced in the modelling as necessary. These account for ∼10% of the weight fraction in the cubic sample and, also based on what was observed for the better crystallized tetragonal sample, are identified as ZnS, cassiterite SnO_2_ and digenite Cu_7.2_S_4_. Following thermal treatments at 833 K, the samples evolved, fully displaying the features of the tetragonal phase, and SnO_2_ and Cu_7.2_S_4_ are clearly detected. TEM/SEM imaging and EDX corroborate the presence of these secondary phases (Note S1): SnO_2_ is found as nanometre-sized (∼10 nm) particles surrounding CZTS grains; multifaceted crystals of Cu sulfide are instead spotted as surface segregations, in accordance with other reports (Ahmad *et al.*, 2015 ▸).

For the modelling of CZTS in samples treated at 833 K, two kesterite fractions, a larger one and a smaller one, have been considered. This hypothesis, aside from providing a better modelling of the peaks, stems from TEM observations of a certain degree of bimodality [see Fig. 4 ▸(*e*)]. For both fractions, the tetragonal disordered (*I* 42*m*) arrangement is employed, together with Gaussian/Lorentzian strain broadening and WPPM-based lognormal distributions to model the domain size. This is chosen as more representative of domain distributions in reality. The coarser phase seems to be the prevailing one, around 2/3 of the sample weight fraction, and with an average domain diameter on the order of 90 nm. The arithmetic mean size for the smaller fraction is refined to ∼30 nm. A possible presence of stacking faults has been considered for the sample treated at 833 K though none of the attempted faulting scenarios have given a significant improvement of the fits: faulting seems to be absent in this case. This result is compatible with the general observation that domain growth promotes the annealing of faults.

Interesting features are noted in the atomic displacement parameters. The DW coefficients (or *B*_iso_) for the cubic phase are refined as larger than for the tetragonal one. Indeed, *B*_iso_ for the cubic sample was refined to 1.88 Å^2^ for the cation site, whereas the tetragonal sample presents a cation-averaged *B*_iso_ of 0.9 Å^2^, in good agreement with literature results (Lafond *et al.*, 2014 ▸). This strong reduction in values, almost half than for the cubic sample, stems presumably from structural disorder. Indeed, *B*_iso_ is proportional to the atomic mean-square displacement (MSD, with 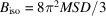 ). Site disorder, by virtue of the increased combination of nearest neighbours and bond lengths, could provide an additional temperature-independent contribution. Moreover, the comparatively smaller domain size of the cubic sample can also be of relevance, as the crystallite surface area was recently shown to contribute in increasing the atomic MSD (Rebuffi *et al.*, 2020 ▸). The connection between atomic disorder and MSD will be thoroughly investigated in Section 3.5. Raman spectra [in Figs. 4 ▸(*c*) and 4(*f*)], owing to their sensitivity to the local atomic environment, would hint at similar observations. Bands are found to be generally broad and with high background, as commonly observed for samples from mechanical alloying (Kapusta *et al.*, 2019 ▸) or any other production technique yielding nanoscale domains (Li *et al.*, 2014 ▸). This can be attributed to a certain complexity of the bonds, defects and disorder in the samples. The tetragonal sample treated at 833 K presents a significant peak sharpening, pointing to increased cation order with respect to the disordered cubic phase. This is also in agreement with the log-Euclidian anisotropy parameter obtained by DFT (Note S6), predicted to be higher for the cubic polymorph.

### 3.4. Temperature evolution

**Figure 7:**
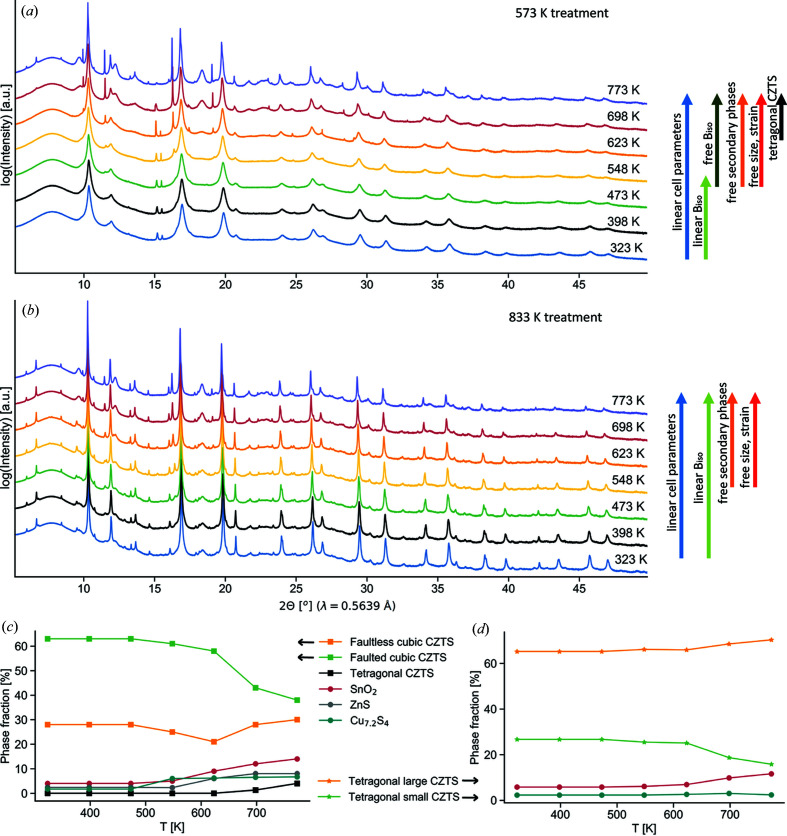
SRXRD over temperature for CZTS samples from mechanical alloying treated at (*a*) 573 K and (*b*) 833 K. Arrows on the right indicate the approach used to refine the data. (*c*) and (*d*) Evolution of phase fractions with temperature from the Rietveld refinement of SRXRD data from (*a*) and (*b*), respectively.

To further examine the effect of structural disorder on the atomic MSD, the temperature evolution of DW coefficients is investigated. Studies over a temperature range are particularly relevant since the interest in the material for applications, especially TEs, involve mid to high operating temperatures. Furthermore, transport properties (especially thermal) have been shown to be crucially dependent on disorder, thus making it meaningful to study the dynamic behaviour of atomic MSD. SRXRD measurements in the range 323–773 K (Fig. 7 ▸) and XRD measurements in the range 100–400 K (Notes S7–S8) are performed for nominally cubic (treated at 573 K) and tetragonal (833 K) samples (Fig. 7 ▸). Specimens differ from those reported in Fig. 3 ▸. Indeed, to allow for a reliable determination of *B*_iso_, a dilution is applied. Nevertheless, Rietveld refinements are performed with the same base models shown before. A simultaneous approach is employed, keeping constant as many parameters as possible for the different patterns in temperature to minimize the number of free variables. A parameter fitting is performed for the lattice parameters and for the DW coefficients of the cation sites by imposing a linear variation with temperature, then left free to vary whenever it is too restrictive (*i.e.* during the cubic to tetragonal phase transition). This approach allows us to increase the robustness of the Rietveld refinement, although it does not prevent the risk of wrong or incomplete constraints leading to systematic deviations. For this reason, attempts by completely freeing lattice parameters and *B*_iso_ have been performed and are detailed in Note S8. A linear relationship is, in most cases, found to suitably model the data. *B*_iso_ of the anion site is set as a free parameter in all the refinements. Variations in the fraction of the secondary phases and in the size and strain components of peak broadening are allowed only above 548 K, where additional/more intense reflections and peak sharpening can be detected. For the sample treated at 573 K, above 623 K some superstructure reflections corresponding to the tetragonal kesterite phase can be spotted, thus an additional *I* 42*m* CZTS fraction has been included in the refinement. This reinforces the observed critical temperature of ∼663 K for the cubic to tetragonal transition by SRXRD (Fig. 2 ▸) and thermal analyses (Isotta *et al.*, 2020*b* ▸).

The evolution of phase fractions can be seen in Figs. 7 ▸(*c*) and 7(*d*). Temperature clearly promotes the development of SnO_2_, Cu_7.2_S_4_ and ZnS secondary phases. Modifications in the CZTS phases can also be observed. For the sample treated at 573 K, the faulted fraction is noted to decrease compatibly with progressive annealing of stacking faults. This effect is partially compensated by an increase in the refined faulting probability (passing from 22 to ∼30%, see Note S8). This feature likely reflects the effect of atomic diffusion and rearrangement from the disordered to the more ordered tetragonal phase. Contextually, the faultless phase fraction seems to remain approximately constant, while the tetragonal phase progressively increases, reaching a maximum of ∼5% at 773 K. Although the last experiment is performed at temperatures above the cubic to tetragonal transition, a full conversion is prevented due to kinetic limitations.

**Figure 8:**
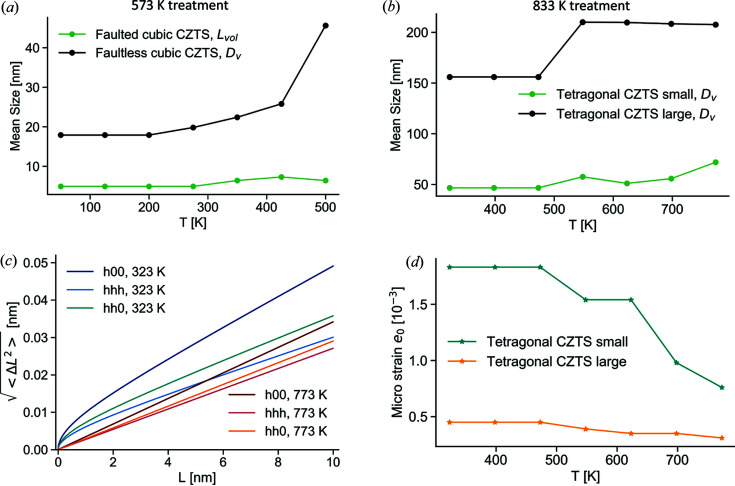
(*a*) and (*b*) Temperature evolution of volume-weighted mean size and (*c*) and (*d*) microstrain from the Rietveld refinement of SRXRD data for samples treated at (*a*) and (*c*) 573 K and (*b*) and (*d*) 833 K. Warren plots in (*c*) refer to the faultless cubic CZTS phase.

For the sample treated at 833 K, the SnO_2_ impurity phase is found to increase. Cu_7.2_S_4_ seems to remain at a constant fraction but experiences grain growth, evident from the corresponding peak sharpening. The larger fraction of tetragonal CZTS is found to increase at the expense of the smaller one. CZTS phases in both samples present generally increasing trends of mean domain size [Figs. 8 ▸(*a*) and 8(*b*)]. This is particularly evident for the sample treated at 573 K, in the highest temperature range (mainly in the faultless fraction), as it is exposed to extreme temperatures. We consistently observed that microstrain [Figs. 8 ▸(*c*) and 8 ▸(*d*)] seems to progressively anneal with temperature, as visible in the decreasing trend of *e*_0_ for the nominally tetragonal sample and in the lowering of the Warren plot curves between 323 and 773 K for the nominally cubic sample (referring to the faultless cubic CZTS phase). The faulted cubic phase was initially refined with *e*_0_ = 0 and, although not likely to be representative of the reality, this parameter remains null throughout the temperature progression.

### 3.5. Evolution of atomic mean-squared displacement: static and dynamic components

**Figure 9:**
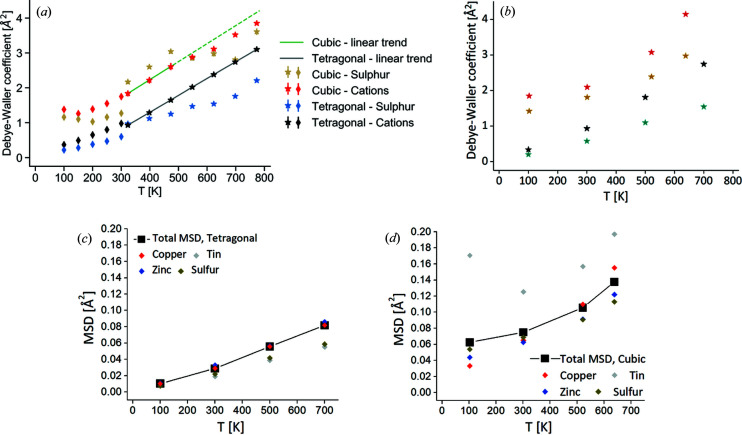
(*a*) Temperature evolution of DW coefficients *B*_iso_ from the Rietveld refinement of high-temperature SRXRD data (star markers) and low-temperature XRD data (diamond markers) for nominally cubic (treated at 573 K) and tetragonal (treated at 833 K) samples. A unique *B*_iso_ was refined for the cation site. See the main text for further details. (*b*) *B*_iso_ for the cubic and tetragonal structures obtained from the MSD calculated from AIMD trajectories. (*c*) MSD of the different ionic species and their average for the ordered tetragonal structure. (*d*) MSD of the different ionic species and their average for the disordered cubic structure.

DW coefficients for the samples treated at 573 and 833 K are visible in Fig. 9 ▸(*a*). For the purpose of comparison, a unique *B*_iso_ value was refined for the different cations of tetragonal CZTS. As expected, the cation *B*_iso_ values increase with temperature, showing good agreement between the low- and the high-temperature datasets. A linear trend models well the cation DW coefficients for the tetragonal sample, whereas for the cubic sample above 500 K *B*_iso_ grows slower than a linear rate. What is most remarkable is the large offset in the trends of the two samples: throughout the temperature range, the cubic sample presents a cation *B*_iso_ almost 1 Å^2^ higher than the tetragonal sample. This points to an additional, temperature-independent contribution which is understood to arise from athermal structural disorder. Full cation disorder in the cubic polymorph leads to a randomization of nearest neighbours, giving rise to inhomogenous bonding and, consequently, a significant distortion of the tetrahedral coordination. This structural disorder, which exists independent of temperature, is known to manifest as an increase in the MSD (Scardi *et al.*, 2017 ▸; Rebuffi *et al.*, 2020 ▸; Mukherjee *et al.*, 2021*a* ▸), thus adding a static component to the *B*_iso_ value (Scardi & Flor, 2018 ▸) above the temperature-dependent vibrational or dynamic contribution. In fact the latter component seems alike for the two samples, as attested by the near-identical slope of the linear trends. Above 500 K, the downwards deviation from the linear trend of the cation *B*_iso_ for the nominally cubic sample is likely to result from a progressive tendency towards order. The DW coefficient of the anion, instead, does not present a systematic trend, with values fluctuating from higher or lower than the cations: we believe these results should be taken with caution, as the ability of diffraction to capture the atomic displacement parameter is reduced with lighter elements. A reliability assessment is presented in Note S9, showing that the *B*_iso_ of the cation possess higher credibility.

To understand the trends in *B*_iso_ from an atomistic point of view, we performed *ab initio* molecular dynamics simulations on both the ordered tetragonal and disordered cubic polymorphs. The MSD of each atomic species is calculated between 100 and 700 K. The MSDs for the disordered polymorph are found to be generally higher than for the tetragonal. For the latter [Fig. 9 ▸(*c*)], the MSDs of the different species cluster together. Cu and Zn ions show slightly higher values, possibly due to their bonding, which is more ionic in nature, and involves lower electron sharing (Isotta *et al.*, 2020*b* ▸). Sn and S, on the other hand, being strongly covalently bound to each other are expected to move the least, and indeed present a comparatively lower MSD. This behaviour is dramatically reversed for the disordered cubic structure [Fig. 9 ▸(*d*)], where Sn dominates the MSD. This can be explained by the *s* ^2^ lone-pair retention and rattling in certain Sn ions (Isotta *et al.*, 2020*b* ▸) [see Figs. 9 ▸(*c*) and 9 ▸(*d*)], shown to possess low-frequency optical modes. These could be responsible for the upward shift in *B*_iso_ and MSD observed for the cubic polymorph. A deviation from linearity at low temperatures is expected, due to the zero-point energy. The presence of disorder-induced vibrational modes surviving at low temperature might explain why this deviation happens at higher temperatures for the cubic phase compared with the tetragonal phase.

By obtaining the weighted *B*_iso_ [Fig. 9 ▸(*b*)] from the calculated MSD we observe good agreement, qualitatively and even somewhat quantitatively, with experiments [Fig. 9 ▸(*a*)]. In particular, the cubic polymorph shows an ∼1 Å^2^ positive offset in the cation *B*_iso_ value. For the tetragonal polymorph, *B*_iso_ shows virtually no static disorder, although in the real sample a non-zero (though minimal) value is expected due to frequently occurring antisites, defects and nanoscale size. From these results, it emerges that the ordered and disordered polymorphs are separated not just by a different vibrational behaviour of the ions (seen in the temperature trend), but also by a distortion of the crystalline lattice due to disorder, seen in the static upward shift in *B*_iso_. The observation of a static component in *B*_iso_ is a further demonstration of cation disorder in the cubic sample. This can add credibility to the assignment of the phase to the disordered sphalerite structure.

## 4. Conclusions

In this work, the novel phase of disordered cubic CZTS from mechanical alloying is carefully studied. The polymorph is metastable at room temperature and is found to undergo transition to tetragonal kesterite above 663 K. Different treatment temperatures are used to produce cubic and tetragonal CZTS samples. Rietveld refinements of SRXRD data allowed us to analyse and compare structural and microstructural features. In particular, the cubic stacking of disordered CZTS is found to possess a considerable fraction of twin faults. These seem to anneal in tetragonal samples, treated at a higher temperature, pointing to an inverse relationship between faulting and domain size. Temperature-dependent SRXRD measurements allow us to observe an upward shift of ∼1 Å^2^ in the cation *B*_iso_ of the cubic sample with respect to the tetragonal counterpart. This is believed to arise from disorder, as the softer bonds and distorted crystalline lattice can accommodate larger atomic MSD. As also confirmed by *ab initio* calculations, disorder leads to a static contribution to MSD, whereas the dynamic component does not differ between the ordered and disordered polymorphs. This work brings further advancement in understanding disorder in CZTS, known to significantly affect thermal and electronic transport properties (Isotta *et al.*, 2020*b* ▸), as well as induce topologically non-trivial behaviour (Mukherjee *et al.*, 2021*b* ▸). DW coefficients obtained from XRD are found to be good indicators of disorder: *B*_iso_ can represent a method to quantify disorder and its dynamic behaviour, of crucial interest to predict and adjust the transport properties. Future work will involve the investigation of possible short-range cation motifs in the disordered cubic arrangement through extended X-ray absorption fine structure and the atomic pair distribution function technique, with the support of *ab initio* modelling.

## Supplementary Material

Supporting figures and tables. DOI: 10.1107/S2052252522000239/fc5058sup1.pdf

## Figures and Tables

**Table 1:** Fit parameters from Rietveld refinement of SRXRD data for samples from mechanical alloying, thermally treated at 573 or 833 K to stabilize the cubic or tetragonal phases, respectively Modelling was performed with the software *TOPAS* (version 7). See the main text for modelling details. Note, as the faulting probability is refined with a separate routine, estimated standard deviations in the table above do not account for the correlation with the parameter of faulting probability.

Sample	Rwp (%)	Phase fractions (wt.%)	CZTS lattice parameters (Å)	Faulting probability (%)	CZTS mean domain size (nm)	Mean strain (-)	Debye–Waller coefficients (Å^2^)
573 K treatment	3.15	29% faultless cubic CZTS, 61% faulted cubic CZTS, 4% SnO_2_, 5% ZnS, 2% Cu_7,2_S_4_	*a* = 5.4150 (1)	22	Faultless WPPM: 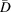 11.6 (4), 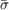 6.2 (3), *D_v_* 18.4 (9) faulted L_Vol_: 5.3 (1)	Faultless WPPM: see Warren plot faulted *e*_0_: ∼0	Cation: 1.88 (1) S: 1.30 (3)

833 K treatment	4.77	66% tetragonal CZTS large, 25% tetragonal CZTS small, 6% SnO_2_, 3% Cu_7,2_S_4_	*a* = 5.4345 (1) *c* = 10.8380 (1)	–	Large WPPM: 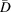 89 (2), 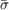 51 (1), *D_v_* 156 (3) small WPPM: 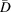 30 (2), 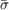 16 (1), *D_v_* 46 (3)	Large *e*_0_: 0.0004 (1) small *e*_0_: 0.0017 (1)	Cation: 0.90 (1) S: 0.66 (1)
