# Supplementary material for: Static and dynamic components of Debye–Waller coefficients in the novel cubic polymorph of low-temperature disordered Cu2ZnSnS4
Source: IUCrJ. 2022 Feb 11;9(Pt 2):272–85. doi: 10.1107/S2052252522000239 (PMC8895019; doi:10.1107/S2052252522000239)
Supplement: Supplementary file 1 [file m-09-00272-sup1.pdf]

IUCrJ

Volume 9 (2022)

Supporting information for article:

Static and dynamic components of Debye–Waller coefficients in the novel cubic polymorph of low-temperature disordered Cu2ZnSnS4

Eleonora Isotta, Binayak Mukherjee, Sebastian Bette, Robert Dinnebier and Paolo Scardi

Static and dynamic components of Debye-Waller coefficients in the novel cubic polymorph of low-temperature disordered Cu_2ZnSnS_4

Supplementary Material

SN 1: Additional TEM and SEM images, and elemental analysis

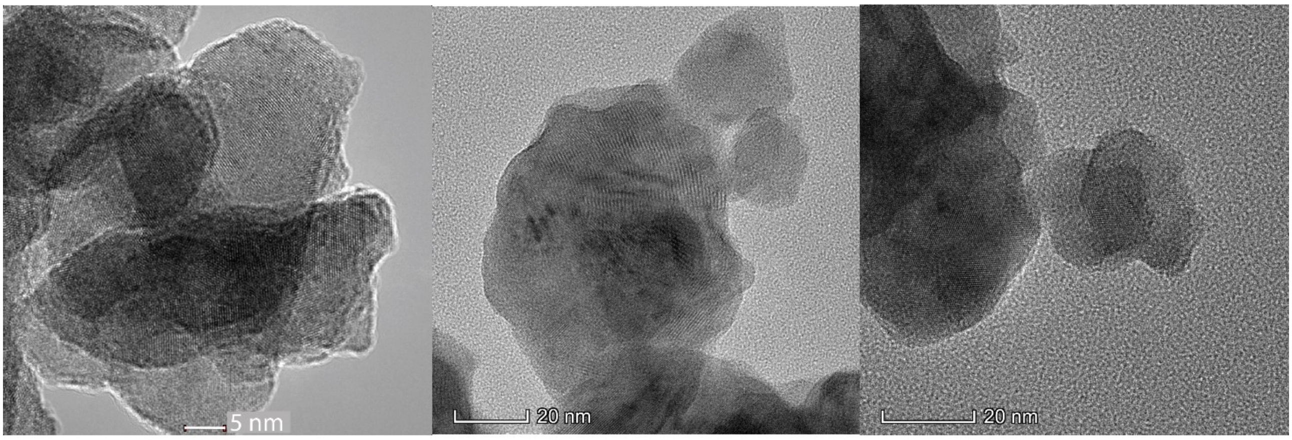

(a) (b) (c)

Figure S1. Transmission electron microscopy images for as-milled CZTS samples from mechanical alloying (a), and treated at 573K (b,c).

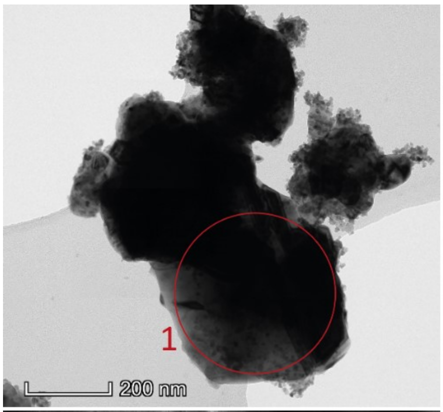

Site	Cu [%at]	Zn [%at]	Sn [%at]	S [%at]	O [%at]
1	33.5	15.7	10.1	40.8	-
2	27.2	14.4	11	47.4	-
3	9.6	3.4	19.4	4.6	62.9
4	5.6	2.6	45.4	5.3	41.2

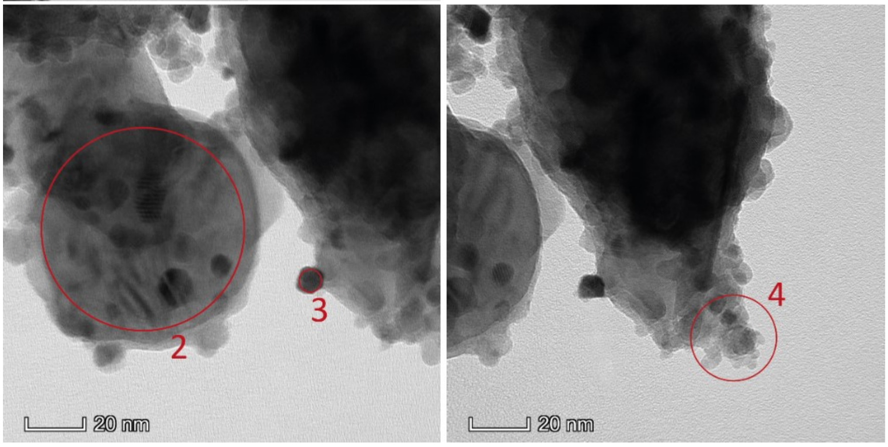

Figure S2. Transmission electron microscopy images and site-specific high-magnification energy dispersive X-ray spectroscopy for a CZTS sample from mechanical alloying treated at 833K. Although the elemental analysis is not calibrated, measurements on areas 1 and 2, likely representing individual domains, show the presence of all the CZTS elements. Areas 3 and 4 clearly show the formation of Sn oxide nanoparticles, as also spotted by XRD. This can explain the Sn depletion observable in the stoichiometry of Areas 1 and 2.

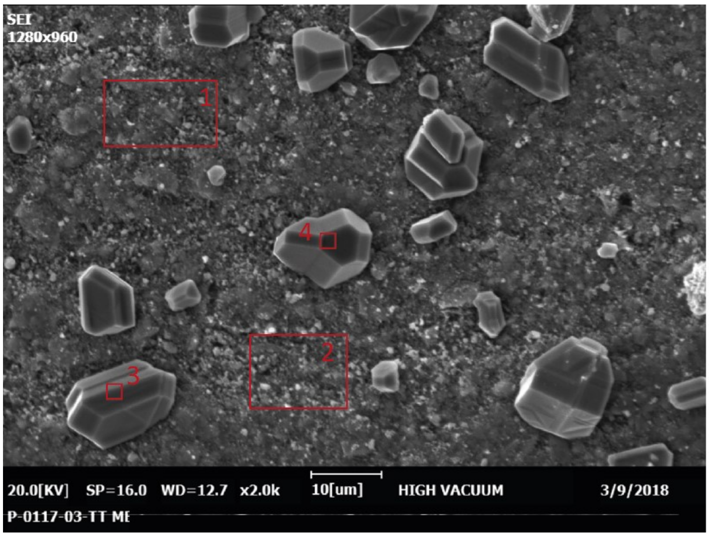

Site	Cu [%at]	Zn [%at]	Sn [%at]	S [%at]
1	27.1	16.7	13.9	42.3
2	26.3	15.3	14	44.4
3	63.4	0.4	0.1	36.1
4	65.9	0.4	0.3	33.3

Figure S3. Scanning electron microscopy with site-specific energy dispersive spectroscopy for a CZTS sample from mechanical alloying treated at 833K (in pellet form). The faceted particles on the surface are identified as segregations of Cu sulphides (probably $Cu_{7.2}S_4$), in accordance with XRD observations.

**Sample**	**Cu/ wt-%**	**Sn/ wt-%**	**Zn/ wt-%**	**S/ wt-%**
theory	28.9	27.0	14.9	29.2
CZTS, as-milled	28.7(1)	26.6(2)	15.3(1)	29.5(5)
CZTS, 573 K treatment	29.2(1)	26.3(1)	15.6(1)	28.9(5)
CZTS, 833 K treatment	29.0(1)	26.4(1)	15.7(1)	29.0(5)

Table S1. Elemental analyses performed with Inductively coupled plasma mass spectrometry for the cation composition and CHNS analysis for the sulphur content.

Table S1 shows the elemental analyses performed with Inductively coupled plasma mass spectrometry for the cation composition and CHNS analysis for the sulphur content. Samples are generally found Sn-poor and Zn-rich, while the composition of Cu is closer to stoichiometric. It is noticed that with the progressively higher thermal treatments a relative loss of sulphur occurs, as expected given the volatile nature of the compound.

SN 2: Additional Raman spectroscopy

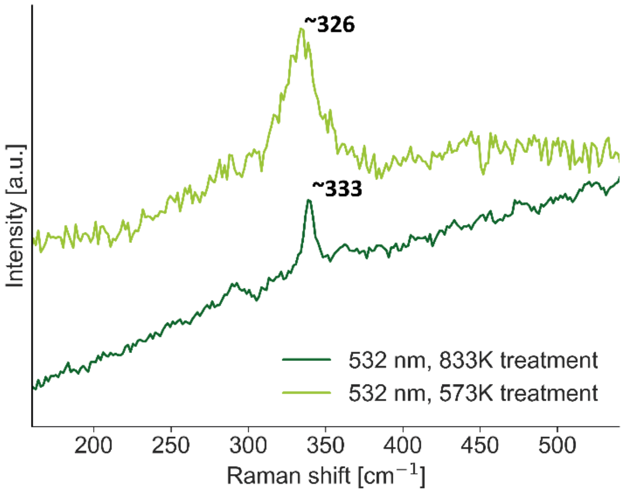

Figure S4. Raman spectra collected with 532nm excitation source.

**Phase**	**Raman scattering peak [cm^{-1}]**	**References**

Table S2. Literature results for Raman scattering peaks of CZTS, CTS and ZnS compounds.

Tetragonal CZTS	289, 339, 350, 370	(Himmrich & Haeuseler, 1991; Altosaar *et al.*, 2008)
Tetragonal CTS	297, 337, 352	(Fernandes *et al.*, 2010)
Cubic CTS	283, 300, 337, 354	(Lohani *et al.*, 2020)
ZnS	275, 352	(Nilsen, 1969)

SN 3: Modelling stacking fault disorder in CZTS

Unit cell transformation

In the crystal structure of cubic CZTS, a stacking of layers of corner sharing (Cu/Sn/Zn)$S_{4/2}$ tetrahedra occurs along the [111] direction. Using DIFFaX-like recursive supercell approaches requires a unit cell transformation (Figure S5) into an either pseudo-orthorhombic or trigonal cell, where the layers are stacked along the crystallographic *c*-axis. The lattice parameters, as well as the atomic coordinates of the transformed unit cell are given in Table S2.

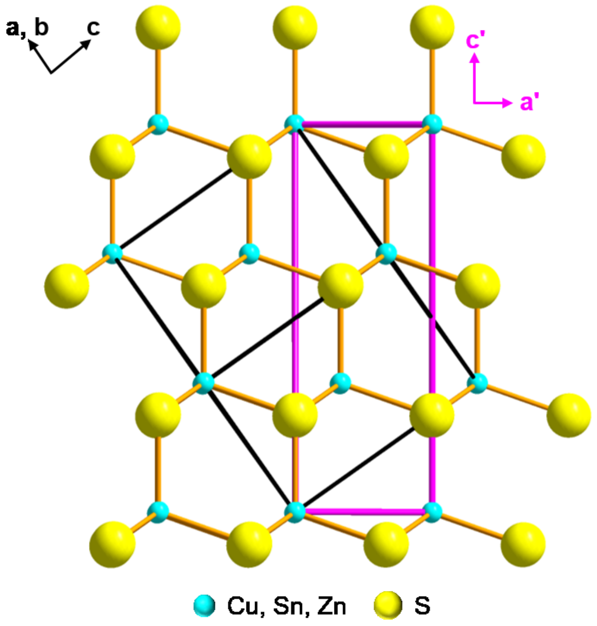

Figure S5. Transformation of the cubic unit cell of CZTS (black lines and coordinate system) into a pseudo trigonal cell (magenta lines and coordinate system). The lattice parameters are shown in Table S2.

	original unit cell			transformed unit cell		
**space group**	*F*$\bar{4}$3*m*			*P*1		
**a/ Å**	5.4196			3.8326		
**b/ Å**	5.4196			3.8326		
**c/ Å**	5.4196			9.3871		
**α/ °**	90			90		
**β/ °**	90			90		
**γ/ °**	90			120		
**V/ Å³**	159.18			119.39		
**Z**	4			3		
**Atomic coordinates**	**x**	**y**	**z**	**x**	**y**	**z**
**Cu/Sn/Zn(1)**	0	0	0	0	0	0

				1/3	2/3	1/3
				2/3	1/3	2/3
**S(1)**	0.25	0.25	0.25	2/3	1/3	-1/12
				0	0	3/12
				1/3	2/3	7/12

Table S3. Unit cell transformation of cubic CZTS used for the refinement of the stacking fault disorder.

Faulting scenarios

The crystal structure of cubic CZTS consists of a cubic close packing of S anions and occupationally disordered Cu, Zn and Sn cations situated in the tetrahedral interstitial voids. The transition from a layer to the subsequent one can be described by using the stacking vector $\overrightarrow{S1}$. Within the faultless structure the stacking occurs in a homogenous fashion, *i.e.* the stacking vector does not change (see **Error! Reference source not found.** in the main text and Figure S6). Twinning describes a prominent faulting scenario for crystals, where differently oriented crystallites are intergrown. For the CZTS cubic structure, this means that the stacking order of the staggered sulfide layers is inverted and that the metal cations occupy a neighboring, tetrahedrally coordinated interstitial void. A single twin consists of two homogenously stacked sections with different stacking vectors ($\overrightarrow{S1}$ and $\overrightarrow{S2}$, Figure S6). In a polycrystalline sample twinning can occur multiple times

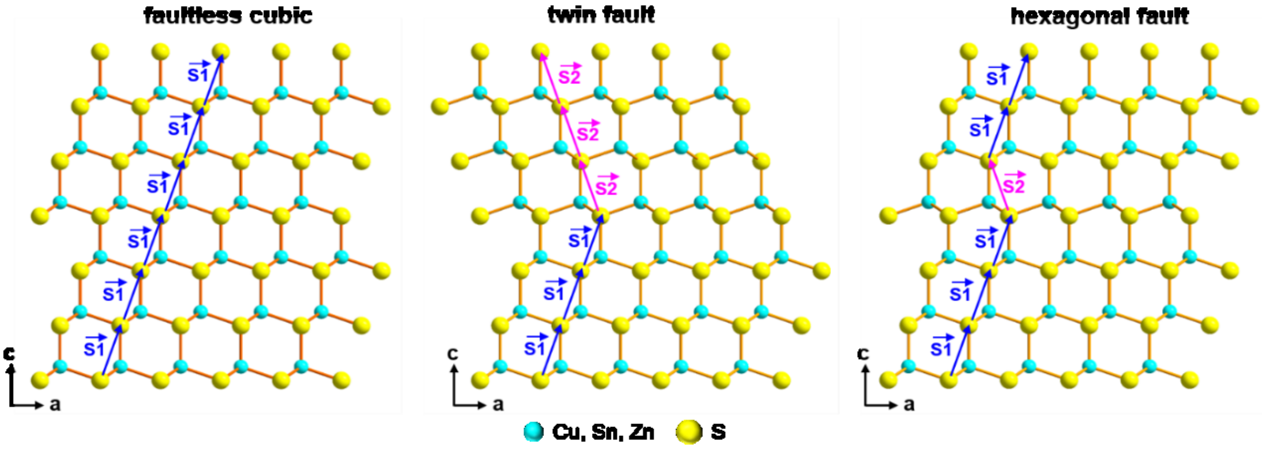

Figure S6. Structural motives considered to occur in the microstructure of cubic CZTS. From left: faultless cubic stacking, cubic stacking with a twin fault, cubic stacking with a hexagonal deformation fault. $\overrightarrow{S1}$ and $\overrightarrow{S2}$ represents the stack

with a random distribution of twin boundaries. An alternating arrangement of differently oriented stacking vectors, i.e. of $\overrightarrow{S1}$ and $\overrightarrow{S2}$ leads to a hexagonal close packing of the anion substructure. This can also occur as an isolated fault, which is comparable to a dislocation in the layer stacking.

For the investigations of the microstructure of cubic CZTS we considered both twin and hexagonal faults. As the metal cations occupy different tetrahedral interstitial voids after an inversion of the stacking order of the anions, two layer types, consisting of one anion and one cation, were defined. The basic faultless cubic stacking was defined by combining layer type A with stacking vector $\overrightarrow{S1}$. A change from layer type A to type B also occurs via the stacking vector $\overrightarrow{S1}$ and is connected to a fault probability (from 0 to 1), px for twin faults and py for hexagonal faults. The probability for a continuation of the faultless stacking order equals to one minus the fault probabilities. In the twin faulting scenario, a stacking of layer type B via stacking vector $\overrightarrow{S2}$ is considered as the new basic stacking order and switching back to layer type A is considered as an additional fault. For a hexagonal fault, the stacking switches immediately (probability = 1) back from layer type B to layer type A. We considered a combination of twin and hexagonal faults as well. A graphical representation of the faulting models is given in Figure S7 and the stacking vectors, layer setups and the transition probability matrixes can be found in Tables S4 and S5.

Atomic coordinates	layer-type A			layer-type B		
**Cu/Sn/Zn(1)**	0	0	0	1/3	2/3	0
**S(1)**	2/3	1/3	-1/12	2/3	1/3	-1/12
**stacking vectors**		$\overrightarrow{S1}=\begin{pmatrix}1/3\\2/3\\1/3\end{pmatrix}$			$\overrightarrow{S2}=\begin{pmatrix}2/3\\1/3\\1/3\end{pmatrix}$	

Table S4. Layer-types and stacking vectors used for the refinement of the stacking fault disorder in cubic CZTS.

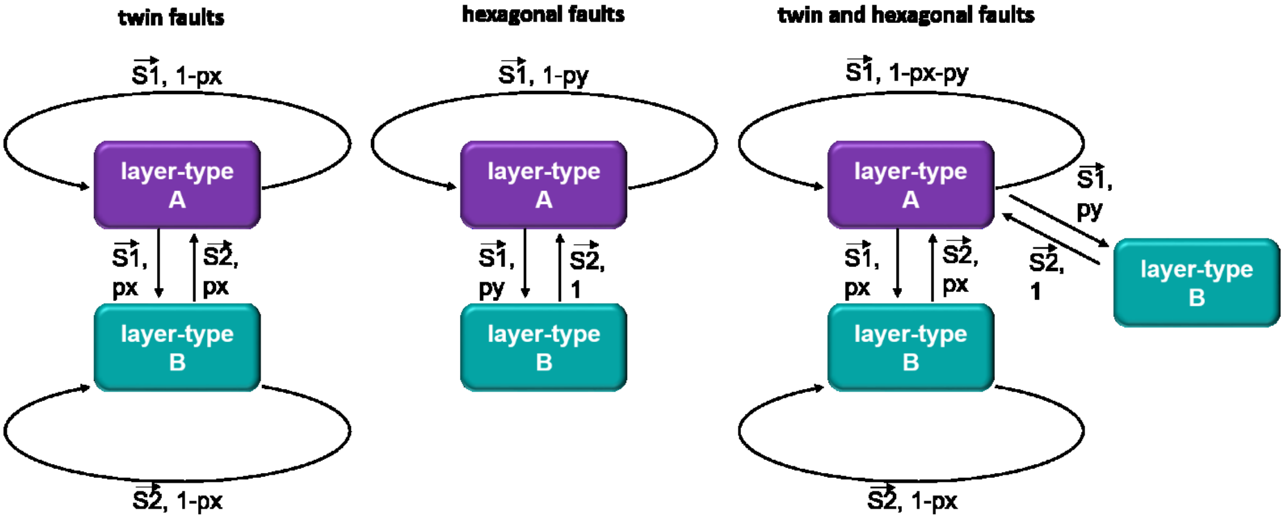

Figure S7. Graphical representation of the faulting scenarios used for describing the microstructure of cubic CZTS.

**twin faults** from↓ to→	layer-type A	layer-type B	**hexagonal faults** from↓ to→	layer-type A	layer-type B
layer-type A	1-px, $\overrightarrow{S1}$	px, $\overrightarrow{S1}$	layer-type A	1-py, $\overrightarrow{S1}$	py, $\overrightarrow{S1}$
layer-type B	px, $\overrightarrow{S2}$	1-px, $\overrightarrow{S2}$	layer-type B	1, $\overrightarrow{S2}$	0, $\overrightarrow{S2}$

twin and hexagonal faults

from↓ to→	layer-type A	layer-type B	layer-type B
layer-type A	1-px-py, $\overrightarrow{S1}$	px, $\overrightarrow{S1}$	py, $\overrightarrow{S1}$
layer-type B	px, $\overrightarrow{S2}$	1-px-py, $\overrightarrow{S2}$	py, $\overrightarrow{S2}$
layer-type B	1, $\overrightarrow{S2}$	0	0

Table S5. Transition probability matrixes used for describing twin and hexagonal stacking faults in cubic CZTS, with px and py being the fault probabilities.

Global and local optimizations

Different faulting scenarios of cubic CZTS were tested using 1D- and 2D-grid search routines. For all tests, possible side products like cassiterite or digenite were excluded. In a first attempt, only one faulted, cubic CZTS phase was used applying either twin or hexagonal faults (Figure S8, filled symbols). The twin faulting scenario resulted in a significant lower R-wp value (6.55 vs. 8.63%, Table S5). As both diffraction line profile analyses with WPPM macros and TEM images indicated a bimodal character of the sample (see **Error! Reference source not found.Error! Reference source not found.** and **Error! Reference source not found.** in the main text), a two phase approach was used for the microstructural refinements as well. Both phases were defined using identical crystallographic parameters, only the strain, crystallite size and the scaling factors were treated individually. One phase was defined as a faultless, cubic CZTS phase and the other phase as faulted CZTS phase using either twin or hexagonal faults. In general, using two phases yielded significantly lower R-wp values (Figure S8, open symbols), with twin faults (Figure S8, open black squares) providing the best result with a fault probability px of 0.22 (Table S5). The simultaneous occurrence of both twin and hexagonal faults was considered as well. Using only one CZTS phase did not provide an improvement in the refinements (Table S5). Using two faulted phases, one twin and one hexagonal faulted phase, slightly improved the R-wp value by 0.08 (Table S5). Nevertheless, we evaluate the faulting scenario consisting of a faultless cubic CZTS phase and a twin faulted CZTS phase as more appropriate as:

- using two faulted phases leads to further extension of the parameter space that is not justified by the poorly significant decrease in the R-wp value
- the fault probability of the hexagonal faulted phase was refined to 0.01, so as almost faultless
- the twin faulting scenario does not exclude hexagonal faults, as the latter materializes as two subsequent twin faults with a probability of px^2. With the refined twin fault probability of 0.22 there is an intrinsic probability for the occurrence of hexagonal faults of approximately 0.04

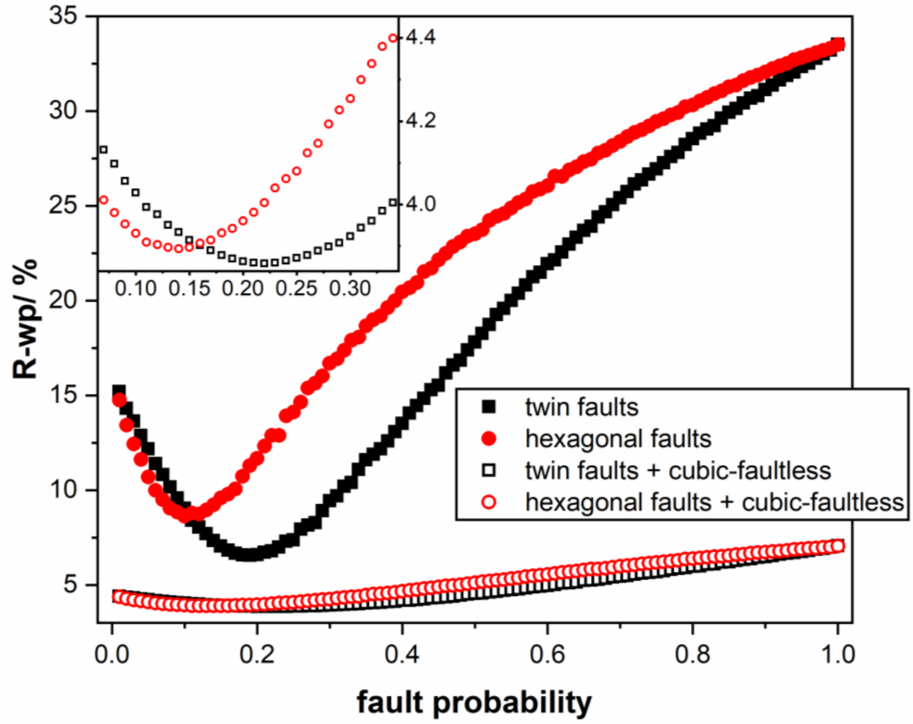

Figure S8. Results of 1-D grid search optimization for the refinement of the XRD pattern of cubic CZTS using different faulting models. The global minimum was found using twin faults and a cubic faultless phase at a fault probability of 0.22 with R-wp = 3.86, without using any other phase.

microstructural model	R-wp/ %	fault probabilities
twin faults [1 phase]	6.55	px = 0.19 py = n.a.
hexagonal faults [1 phase]	8.63	px = n.a. py = 0.10
twin faults + cubic faultless [2 phases]	3.86	px = 0.22 py = n.a.
hexagonal faults + cubic faultless [2 phases]	3.89	px = n.a. py = 0.14
twin faults + hexagonal faults [1 phase]	3.79	px = 0.26 py = 0.01
twin faults + hexagonal faults [2 phases]	6.52	px = 0.18 py = 0.01

Table S6. Overview of the investigated faulting models, including the fault probabilities (Table S4, Figure S6) and the associated R-wp at the global minima of the grid search routines.

SN 4: TOPAS structure files for cubic sphalerite CZTS and faulted cubic CZTS

```
str
space_group F-43m
phase_name "Cubic sphalerite CZTS"
' Formula: Cu2 Zn Sn S4
a 5.4150
b 5.4150
c 5.4150
al 90
be 90
ga 90
site Zn    x 0.00000    y 0.00000    z 0.00000    occ Zn 0.25    beq 1.88
site Sn    x 0.00000    y 0.00000    z 0.00000    occ Sn 0.25    beq 1.88
site Cu    x 0.00000    y 0.00000    z 0.00000    occ Cu 0.5     beq 1.88
site S     x 0.25000    y 0.25000    z 0.25000    occ S 1        beq 1.30
' Unit cell volume [A^3]: 158.782
' Density (calculated) [g/cm^3]: 4.596
scale sc 0.000148342352`
```

```
' WPPM model for size, lognormal distribution of spherical domains:
prm mu  2.32579`_0.03221 min 0.5 max = Min(2 Val + .02, 5); val_on_continue = Rand(1, 3);
prm si  0.49999`_0.01041 min .1 max = Min(2 Val + .01, 0.5); val_on_continue = Rand(.1, 1);
prm !Diameter = Exp(mu+si^2/2); :  11.59745`_0.37838
rm !StanDev = (Exp(2*mu+si^2)*(Exp(si^2)-1))^(1/2); :  6.18061`_0.26680
prm !DiameterVol = 3/4*Exp(mu+7/2*si^2); :  18.41329`_0.89515

' WPPM PAH model for general microstrain, cubic symmetry:
prm !av  1.43234 min 0 val_on_continue = Rand(0, 0.1);
prm !bv  1.59045 min 0 val_on_continue = Rand(0, 0.1);
prm !E1val  0.78265 min 0 val_on_continue = Rand(0, 0.1);
prm !E2val  0.03021  min 0
WPPM_Strain_InvariantPAH(   , av,   , bv, 216, E1val,E2val )


str
space_group P1
phase_name "Faulted cubic CZTS"
' Formula: Cu2 Zn Sn S4
' Density (calculated) [g/cm^3]: 4.591
a 3.8290
b 3.8290
c = Get(generated_c);
al 90
be 90
ga 120
prm !h1  3.13003
prm !os  0.23109
prm !b1  1.87710`_0.01025 min 0.1 max 5
prm !b2  1.29781`_0.02666 min 0.1 max 5
prm !py  0.22 'Faulting probability
generate_stack_sequences {
        pr_str { B layers_tol} 1000
        number_of_sequences Nstr 100
        number_of_stacks_per_sequence Nv 500
        Transition(A, h1)
                to A = 1-py; a_add =  1/3; b_add = 2/3;
                to B = py;   a_add =  1/3; b_add = 2/3;
        Transition(B, h1)
                to A = py;    a_add =  2/3; b_add = 1/3;
                to B = 1-py; a_add =  2/3; b_add = 1/3;
}
        site Zn1  x =  0.0000 ; y = 0.0000 ; z  = (0/Nv);        occ Zn     0.25  beq  = b1; layer A
        site Cu1  x =  0.0000 ; y = 0.0000 ; z  = (0/Nv);        occ Cu     0.50  beq  = b1; layer A
        site Sn1  x =  0.0000 ; y = 0.0000 ; z  = (0/Nv);        occ Sn     0.25  beq  = b1; layer A
        site S1   x =  2/3;     y = 1/3;     z  = ((os*3)-1)/Nv;  occ S      1     beq  = b2; layer A

        site Zn1  x =  1/3;     y = 2/3;     z  = (0/Nv);        occ Zn     0.25  beq  = b1; layer B
        site Cu1  x =  1/3;     y = 2/3;     z  = (0/Nv);        occ Cu     0.50  beq  = b1; layer B
        site Sn1  x =  1/3;     y = 2/3;     z  = (0/Nv);        occ Sn     0.25  beq  = b1; layer B
        site S1   x =  2/3;     y = 1/3;     z  = ((os*3)-1)/Nv;  occ S      1     beq  = b2; layer B
```

peak_buffer_based_on = Xo;
peak_buffer_based_on_tol 0.1
scale = sc / (Nv Nstr);

SN 5: Ground-state binding energy for the different structures

	**Binding energy per unit atom [eV]**
**Sphalerite $F\bar{4}3m$ CZTS**	-4.02
**Wurtzite $P6_3mc$ CZTS**	-3.98

Table S7. Ground-state binding energies per unit atom calculated ab initio for a CZTS cubic sphalerite and a CZTS hexagonal wurtzite structure.

SN 6: Compliance tensors and anisotropy in CZTS

In order to measure the anisotropy in CZTS, we performed DFT calculations using the finite difference method. This allows us to obtain the compliance tensor for the ordered and disordered polymorphs, which are given respectively as,

$$C_{ordered} = \begin{bmatrix} 97.9 & 59.5 & 58.7 & 0.3 & 0 & 0 \\ 59.5 & 97.9 & 58.7 & -0.3 & 0 & 0 \\ 58.7 & 58.7 & 96.3 & 0 & 0 & 0 \\ 0.3 & -0.3 & 0 & 43.2 & 0 & 0 \\ 0 & 0 & 0 & 0 & 43.8 & 0 \\ 0 & 0 & 0 & 0 & 0 & 43.8 \end{bmatrix}$$

and

$$C_{disordered} = \begin{bmatrix} 55.7 & 40.5 & 41.3 & -2.9 & 4.6 & 3.8 \\ 40.5 & 50.2 & 44.4 & 6.2 & 7.7 & 2.9 \\ 41.3 & 44.4 & 63.9 & -2.4 & 2.1 & 1.5 \\ -2.9 & 6.2 & -2.4 & 12.6 & -1.3 & -2.5 \\ 4.6 & 7.7 & 2.1 & -1.3 & 11.8 & -10.5 \\ 3.8 & 2.9 & 1.5 & -2.5 & -10.5 & 2.8 \end{bmatrix}$$

where all values are in GPa.

It should be noted that to minimize the forces acting on individual ions, the DFT reduces the symmetries of the supercell to triclinic. This leads to small non-zero values of c_{ij} for $i,j > 3$ ($i \neq j$). In addition, given the metastable nature of the disordered polymorph, several negative terms are also introduced. Nevertheless, this does not significantly affect the overall qualitative nature of the anisotropy in the two polymorphs, or their comparison. For the ordered tetragonal polymorph, the compliance matrix is in good agreement with that obtained by He and Shen(He & Shen, 2011).

In order to quantify the degree of anisotropy, we use the log-Euclidean anisotropy parameter A^L (Kube, 2016), which is valid for all crystalline symmetries. It is an absolute measure of anisotropy, with perfect isotropy yielding a value of zero. This measure of anisotropy is given as,

$$A^L(C^V, C^R) = \sqrt{\left[ln\left(\frac{\kappa^V}{\kappa^R}\right)\right]^2 + 5\left[ln\left(\frac{\mu^V}{\mu^R}\right)\right]^2}$$

where C is the compliance tensor, κ and μ are the bulk and shear moduli respectively, while the superscripts V and R refer to the Voigt and Reuss averages.

For the ordered tetragonal polymorph, this parameter has a value of 0.77 while for the disordered cubic this comes out to 1.12, indicating that the latter polymorph is more anisotropic. This of course is in agreement with the fact that full cation disorder leads to significant inhomogeneous bonding in cubic CZTS. In comparison to $SrVO_3$, which is the most anisotropic cubic crystal shown in Ref #, with a value of 5.3, CZTS presents a relatively limited anisotropy.

In order to further understand the nature of the anisotropy, we have calculated the Young's modulus projected along the [hhh] and [h00] family of directions. The reciprocal of Young's Modulus in the direction of the unit vector l_i for the general triclinic system is given by (Nye, 2006),

$$l_1^4 s_{11} + 2l_1^2 l_2^2 s_{12} + 2l_1^1 l_3^2 s_{13} + 2l_1^2 l_2 l_3 s_{14} + 2l_1^3 l_3 s_{15} + 2l_1^3 l_2 s_{16}$$

$$+l_2^4 s_{22} + 2l_2^2 l_3^2 s_{23} + 2l_2^3 l_3 s_{24} + 2l_1 l_2^2 l_3 s_{25} + 2l_1 l_2^3 s_{26}$$

$$+l_3^4 s_{33} + 2l_2 l_3^3 s_{34} + 2l_1 l_3^3 s_{35} + 2l_1 l_2 l_3^2 s_{36}$$

$$+l_2^2 l_3^2 s_{44} + 2l_1 l_2 l_3^2 s_{45} + 2l_1 l_2^2 l_3 s_{46}$$

$$+l_1^2 l_3^2 s_{55} + 2l_1^2 l_2 l_3 s_{56}$$

$$+l_1^2 l_2^2 s_{66}$$

where s_{ij} are the elements of the stiffness tensor.

We represent the [hhh] direction by taking the average of all [111] directions, while the [h00] direction is represented by the average of [100], [010] and [001] directions. For the ordered tetragonal, the average reciprocal Young's modulus for the [hhh] directions is obtained as 0.0092 GPa^{-1} (E = 108.7 GPa) while for the [h00] directions the value is 0.019 GPa^{-1} (E = 52.6 GPa). Similarly, for the disordered cubic, along the [hhh] directions we get a value of 0.02 (E = 50 GPa), while along the [h00] directions the value is 0.04375 (E = 22.9 GPa).

These results indicate that for both the ordered and disordered polymorphs of CZTS, the [hhh] direction is stiffer in comparison to the [h00] direction. This can be explained by the fact that the rigid Sn – S covalent bonds lie along the 111 directions, making deformation along that direction significantly more difficult than in the 100 direction.

SN 7: Low-temperature XRD data

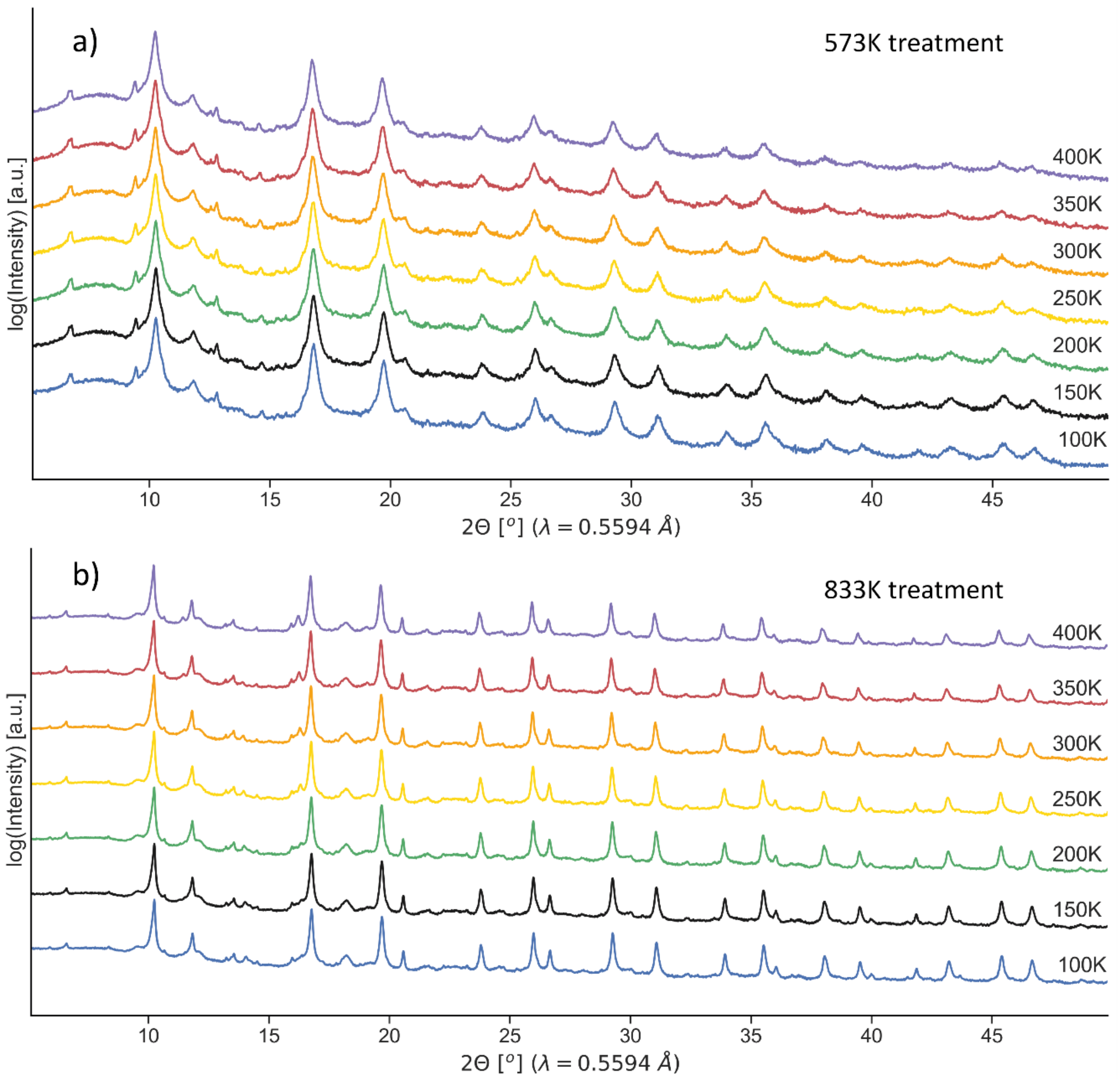

Figure S9. XRD in temperature for CZTS samples from mechanical alloying treated at 573K (a) and 833K (b).

SN 8: Further data about Rietveld refinements of XRD patterns in temperature

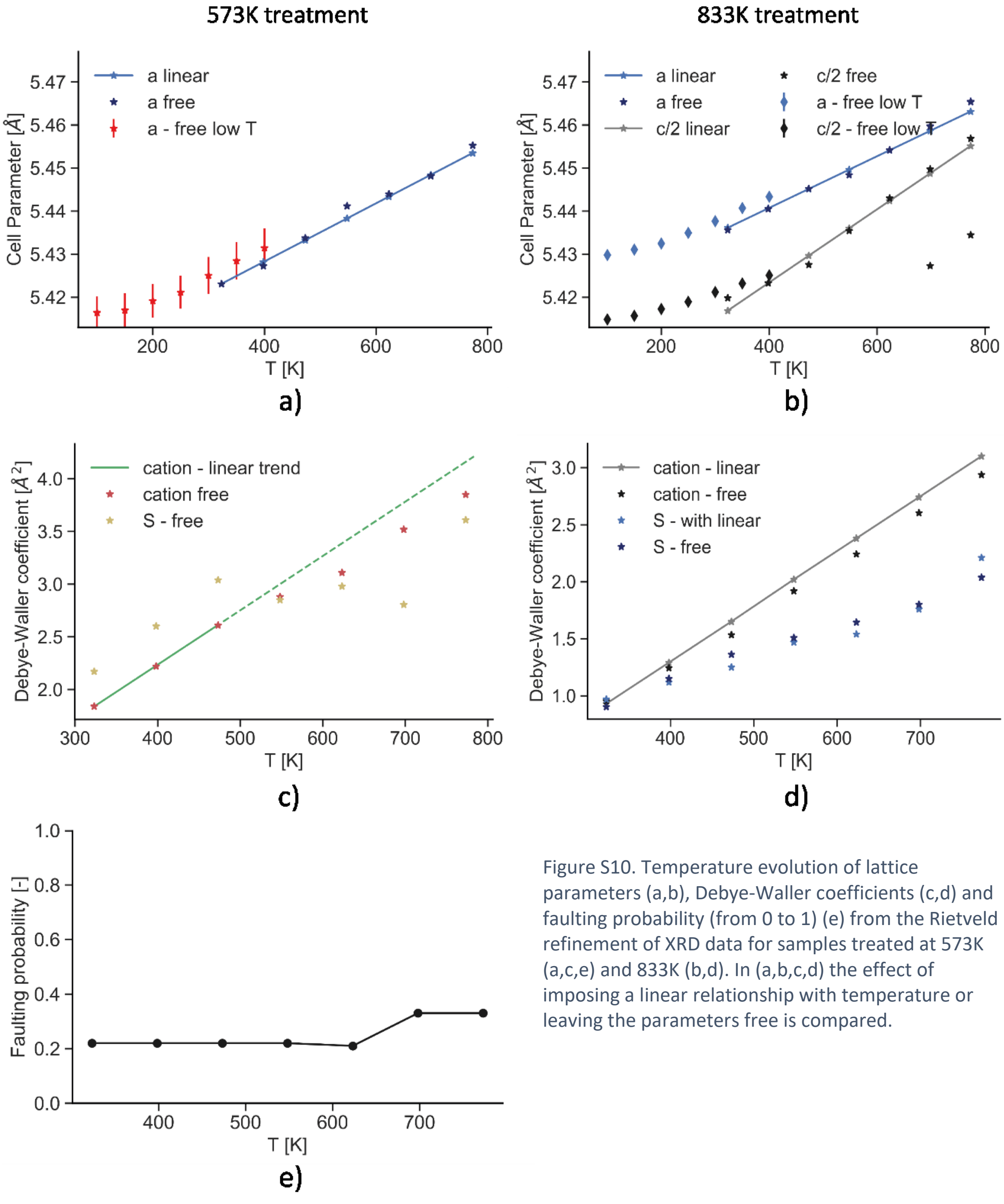

Figure S10. Temperature evolution of lattice parameters (a,b), Debye-Waller coefficients (c,d) and faulting probability (from 0 to 1) (e) from the Rietveld refinement of XRD data for samples treated at 573K (a,c,e) and 833K (b,d). In (a,b,c,d) the effect of imposing a linear relationship with temperature or leaving the parameters free is compared.

SN 9: Reliability of Debye-Waller coefficients

Figure S9 shows a comparison of reliability between B_{iso} values for the cation and the anion. The trend of the refinement Rwp for an imposed variation in B_{iso}, clearly shows that the fit is less sensitive towards a variation in the anion B_{iso}. The minima are instead more clearly identified for the cation B_{iso}. Furthermore, the estimated standard deviations (known to be an underestimation of the real value error) are found between 2 to 3 times larger for the anion's value than the cation's. These features, coupled with the known lower accuracy of XRD in determining the atomic displacement parameter for lighter elements, point to a much-reduced credibility of the anion values. This can explain the stronger fluctuation of experimental values which is observed between different measurements and datasets. In addition, we do not exclude possible sample deviations between the high-and the low-temperature datasets, as the specimens were different.

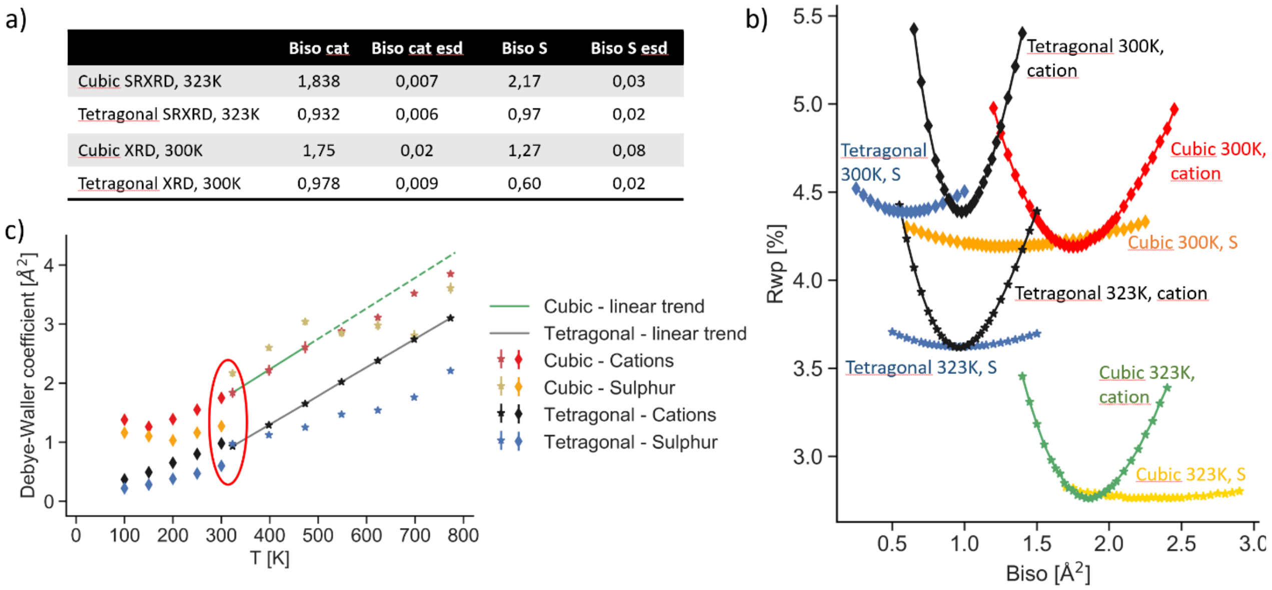

a)

	Biso cat	Biso cat esd	Biso S	Biso S esd
Cubic SRXRD, 323K	1,838	0,007	2,17	0,03
Tetragonal SRXRD, 323K	0,932	0,006	0,97	0,02
Cubic XRD, 300K	1,75	0,02	1,27	0,08
Tetragonal XRD, 300K	0,978	0,009	0,60	0,02

Figure S11. a) Debye-Waller coefficients B_{iso} and corresponding estimated standard deviation esd from Rietveld refienement. b) Trend of the refinement Rwp with imposed variations of Biso. Data in a) and b) refer to SRXRD data collected at 323K and XRD data collected at 300K, as highlighted in red in panel c).

References

Altosaar, M., Raudoja, J., Timmo, K., Danilson, M., Grossberg, M., Krustok, J. & Mellikov, E. (2008). *Phys. Status Solidi Appl. Mater. Sci.* **205**, 167–170.

Fernandes, P. A., Salomé, P. M. P. & Da Cunha, A. F. (2010). *Phys. Status Solidi Curr. Top. Solid State Phys.* **7**, 901–904.

He, X. & Shen, H. (2011). *Phys. B Condens. Matter*. **406**, 4604–4607.

Himmrich, M. & Haeuseler, H. (1991). *Spectrochim. Acta Part A Mol. Spectrosc.* **47**, 933–942.

Kube, C. M. (2016). *AIP Adv.* **6**, 1–6.

Lohani, K., Isotta, E., Ataollahi, N., Fanciulli, C., Chiappini, A. & Scardi, P. (2020). *J. Alloys Compd.* **830**, 154604.

Nilsen, W. G. (1969). *Phys. Rev.* **182**, 838–850.

Nye, J. F. (2006). Physical Properties of Crystals Oxford.
